# Subtropical adaptation of a temperate plant (*Brassica oleracea* var. *italica*) utilizes non-vernalization-responsive QTLs

**DOI:** 10.1038/s41598-018-31987-1

**Published:** 2018-09-11

**Authors:** Yann-rong Lin, Jou-yi Lee, Meng-chun Tseng, Chieh-ying Lee, Chian-he Shen, Chun-shan Wang, Chia-ching Liou, Lan-shuan Shuang, Andrew H. Paterson, Kae-kang Hwu

**Affiliations:** 1 Department of Agronomy, National Taiwan University, Taipei, 10617 Taiwan; 2 Department of Horticulture, Chiayi Agricultural Experiment Station, Taiwan Agricultural Research Institute, Chiayi, 60044 Taiwan; 3 Known-You Seed Co., LIR, Kaohsiung, 84043 Taiwan; 4 Plant Genome Mapping Laboratory, University of Georgia, Athens, Georgia 30602 USA

## Abstract

While many tropical plants have been adapted to temperate cultivation, few temperate plants have been adapted to the tropics. Originating in Western Europe, *Brassica oleracea* vernalization requires a period of low temperature and *BoFLC*2 regulates the transition to floral development. In *B. oleracea* germplasm selected in Taiwan, a non-vernalization pathway involving *BoFLC3* rather than *BoFLC*2 regulates curd induction. In 112 subtropical breeding lines, specific haplotype combinations of *BoFLC3* and *PAN* (involved in floral organ identity and a positional candidate for additional curd induction variation) adapt *B. oleracea* to high ambient temperature and short daylength. Duplicated genes permitted evolution of alternative pathways for control of flowering in temperate and tropical environments, a principle that might be utilized via natural or engineered approaches in other plants. New insight into regulation of Brassica flowering exemplifies translational agriculture, tapping knowledge of botanical models to improve food security under projected climate change scenarios.

## Introduction

*Brassica oleracea* (CC genome, 2n = 18), originating from coastal areas of Western Europe, has been domesticated and selected for diverse morphological variation to form important crops including broccoli, Brussels sprouts, cabbage, cauliflower, kale, and others. Broccoli (var. *italica*), cultivated for its thickened edible inflorescence^1^, has also gained attention for containing rich anticancer compounds, glucosinolates^2^ and demand is increasing globally. However, broccoli production is restricted to limited areas or cool seasons because its flowering is triggered by facultative vernalization and curds properly form under low temperature. High temperature impedes differentiation of floral meristems and results in uneven-sized floral buds^3,4^. To adjust flowering time to optimize broccoli production will support food security and food resilience. Characteristics of non-vernalization and heat tolerance are compulsory for broccoli cultivation under global warming, and for extending cultivation to subtropical zones.

Flowering time (FTi), the consequence of the transition from the vegetative stage to the reproductive stage of growth, is genetically regulated; is highly responsive to environmental cues; and is a prime trait for plant adaptation to diverse geographic regions and seasons^5,6^. In addition, FTi is of central importance to determining crop cultivation and harvest seasons to optimize yield and quality. More than 300 flowering genes have been identified in the model plant, *Arabidopsis thaliana*, and genetically characterized into five main pathways, converging on two major floral integrators, FLOWERING LOCUS T (FT) and SUPPRESSOR OF OVEREXPRESSION OF CO1 (SOC1), which transition meristem identity from shoot apical meristems to inflorescence meristems^5,7–9^. The gene expression of both *FT* and *SOC1* is supressed by FLOWERING LOCUS C (FLC), which expression is reduced by vernalization, a long duration of low temperature^10^. Since *Arabidopsis* and *Brassica* are members of the Brassicaceae family, their genetic pathways of flowering tend to be conserved, with orthologous genes for flowering time/curd initiation time, and substantial vernalization needed to promote flowering^11–13^. However, flowering genes, like other *Brassica* genes, occur in multiple copies as a result of whole genome triplication after its divergence from a common ancestor shared with *Arabidopsis*^14^. For instance, only one *FLC* gene was identified in *Arabidopsis* versus four, four, and five in *B. oleracea, B. rapa* (AA genome, 2n = 20), and *B. napus* (AACC, 2n = 38), respectively^12,15,16^. Presence/absence variation of flowering genes increases genetic diversity for adaptation to a wide range of climatic zones and latitudes^17,18^.

To identify genes/QTLs affecting flowering time is important to investigating mechanisms underlying the vegetative-reproductive transition in plants, and also can provide information and tools to accelerate marker-assisted selection (MAS) to breed new varieties and broaden cultivation seasons and areas. QTL mapping combined with a candidate gene approach to isolate homologous flowering genes is popular in *Brassica* because of colinearity with *Arabidopsis*^13,19,20^. Numerous studies using different mapping populations, revealed conserved QTLs across different populations and environments^12,13,21–26^. The most conserved QTL on chromosome O2 was inferred to be *BoFLC2*, a vernalization responsive gene in dosage dependent regulation of flowering time with different *BoFLC2* alleles accounting for flowering time variation^11,12,23,27^.

Early curd induction and heat tolerance are important traits for broccoli and cauliflower breeding in Taiwan and other Southeast Asian countries^28^, and a breeding program to develop heat-tolerant broccoli hybrids adapted to the eastern United States was launched in the early 1990s^29^. QTLs conferring days to curd initiation (DCI) are influenced by ambient temperature, as exemplified by only 2 QTLs were consistently detected in a doubled haploid (DH) population and a commercial diversity panel of cauliflower, respectively, at several temperature regimes^22,26^. *BoVRN2* and *BoFLC2* were not related to temperature-regulated curd induction, and suppression of *BoCAL*, *BoAP1-a*, and *BoLFY* or failure to suppress *BoTFL1* could maintain arrest of the inflorescence meristem but not floral initiation^26,30^. The genetic flowering pathway of curd induction and formation in response to temperature, especially to high temperature, is still obscure. We used advanced breeding lines selected under the subtropical environment, relative hot and high humidity, to unveil more genes/QTLs provide new insights into flowering time and curd formation which can be applied to the breeding of broccoli and other Brassica crops.

## Results

### QTL conferring days to curd induction and curd quality

**Figure 1:**
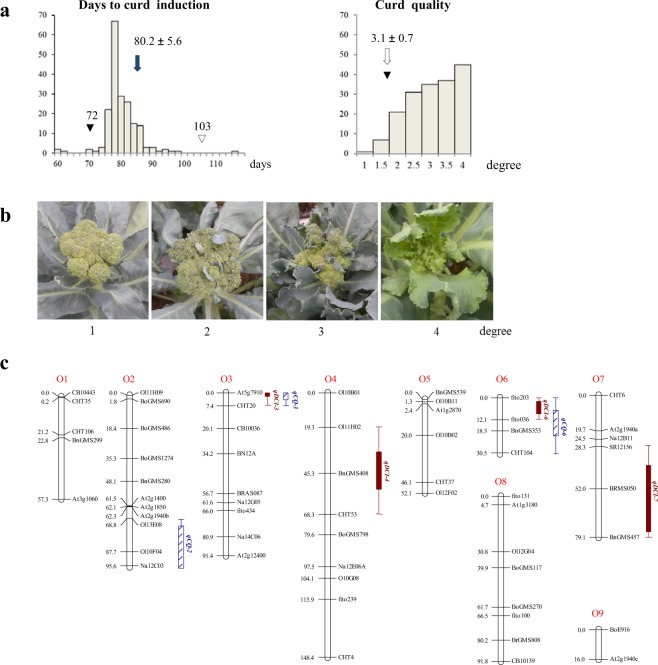
The Phenotypic evaluation and QTL intervals of days to curd induction (DCI) and curd quality (CQ). (**a**) The frequency distributions of DCI and CQ in the F_2_ population. The DCI and CQ of BLM 29 and BLM 25 are indicated by solid (▼) and open (▽) triangles, respectively, and the means of DCI and CQ of the F_2_ population are indicated by arrows (⇧). BLM25 with long DCI failed to initiate curd formation. (**b**) The evaluation of curd quality. The curd quality of broccoli was scored as 1 to 4 according to floral development and abundance of bracts in the leafy curd, from left to right. (**c**) The QTL intervals are indicated by bars and whiskers for 90 and 99% likelihood, respectively, and solid and striped bars are for QTLs conferring DCI and CQ, respectively. SSR markers are indicated with prefixes of BnGMS, Ol, BoGMS, fito, BRMS, Na, CB, BRAS, SORA, BrGMS, and CHT, and IP markers are indicated with prefix of At.

From 112 breeding lines selected under high temperature and humidity by Known-You Seed Co. in Taiwan, an early-maturing kale-derived broccoli (BLM29) was chosen for crossing with a late elite broccoli (BLM25), with the parents and 188 F_2:3_ families evaluated at Tainan, Taiwan (23.079337°N, 120.295377°E) under daily temperatures ranging from 24.5–30.4 °C with a 28.8 °C average. Days to curd induction (DCI) of the parents were 72 and 103 days, while F_2_ progeny averaged 80.2 (±5.6) days and ranged from 59.1 to 117.0 days, with three individuals exhibiting earlier DCI than BLM29 (Fig. 1a). Curd quality (CQ) was rated from 1 to 4 based on the abundance of leafy bracts between inflorescence internodes **(**Fig. 1b). While late-flowering BLM25 was heat sensitive and failed to initiate curds, early-flowering BLM29 formed curds with leafy bracts, rated CQ degree 3. The average CQ of the 177 survivors was 3.1 (±0.37, Fig. 1a), with only one having ideal CQ of 1, but 60 with better CQ than BLM29.

Using a genetic map of 60 well-spaced DNA markers forming nine linkage groups spanning 622.2 cM (Fig. 1c; the primary genetic linkage map with 126 markers is Supplementary Fig. 1), DCI QTLs were identified on chromosomes O3, O4, O6, and O7, explaining 4.03% to 28.54% of phenotypic variance (PV) (Table 1; Fig. 1c). Significant digenic interactions were detected for *qDCI-6* with both *qDCI-3* and *qDCI-7*. A full model, including 4 QTLs and two digenic interactions, explained 55.28% of PV (Table 1). The alleles of early parent BLM29 promoted DCI by 3.81 and 2.07 days for *qDCI-6* and *qDCI-7*, respectively; postponing DCI by 3.80 and 1.78 days for *qDCI-3* and *qDCI-4*, respectively. CQ QTLs were detected on chromosomes O2, O3, and O6 (Table 1; Fig. 1c). The major QTL, *qCQ-6*, mapped to the fito203/CHT104 interval, showing an additive −0.44 degree reduction of curd leafiness by the BLM29 allele, and a full model with the 3 identified QTLs explained 33.02% of PV. The BLM29 allele for *qCQ-2* and *qCQ-6*, but not *qCQ-3*, increased curd quality.

**Table 1:** QTL parameters of days to curd induction and curd quality in broccoli.

Trait	locus	Chr.	Peak position	Marker interval	LOD	Additive	Dominant	PVE%^b^
			(cM)			effect^a^	effect	
DCI	*qDCI-3*	3	0	At5g7910/CHT20	16.07	3.80	−0.13	21.56
	*qDCI-4*	4	44	Ol11H02/CHT53	3.52	1.78	−0.03	4.03
	*qDCI-6*	6	6	fito203/fito036	20.15	−3.81	−2.68	28.54
	*qDCI-7*	7	63	SR12156/BnGMS457	5.62	−2.07	−1.35	6.61
Model^c^ Y = Q1 + Q2 + Q3 + Q4 + Q1:Q3 + Q3:Q4					32.85			55.28
CQ	*qCQ-2*	2	92	Ol13E08/Na12C03	4.95	−0.32	0.13	9.15
	*qCQ-3*	3	0	At5g7910/CHT20	6.44	0.41	−0.07	12.14
	*qCQ-6*	6	15	fito203/CHT104	7.11	−0.44	−0.17	13.52
Model Y = Q1 + Q2 + Q3					15.49			33.02

^a^The magnitudes of additive effects were contributed from the allele of the early parent, BLM29, on days to curd induction (DCI) and curd quality (CQ).

^b^PVE is the abbreviation of phenotypic variance.

^c^Model indicates the full model after forward QTL mapping analysis by using R/qtl. Q1:Q3 and Q3:Q4 are digenic interactions of *qDCI-3* with *qDCI-6* and *qDCI-6* with *qDCI-7*.

The two major DCI and CQ QTLs coincided with one another, mapping to chromosomes O3 and O6 (Table 1; Fig. 1c), implying that these two QTL pairs might each be due to single genes with pleiotropic effects, or different tightly linked genes. The additive effects of these two QTL pairs contrasted in direction–elite BLM25 conferred advantageous alleles *qDCI-3* and *qCQ-3* for early curd induction with good curd formation. BLM29, with early DCI and heat-insensitivity, contributed allele(s) of *qDCI-6* promoting curd induction by 3.81 days; and *qCQ-6* reducing curd leafiness by 0.44 degrees.

To validate their effects, *qDCI-6*/*qCQ-6* alleles of BLM29 were introgressed into BLM25–four BC_3_F_1_ individuals possessed 91.1%, 94.5%, 94.5%, and 94.5% of BLM25 DNA marker alleles and horticultural traits closely resembling BLM25 but with DCIs of 42, 42, 50, and 50 days, much earlier than BLM25 (64 days) (Supplementary Figs 2, 3). In 113 BC_2_F_2_ families, the average DCI of BLM25 homozygotes, 48.9 (±4.6), was significantly later than BLM29 homozygotes and heterozygotes, 39.8 (±5.2) and 42.3 (±5.2) (Supplementary Table 1). In 87 BC_3_F_2_ families, the DCIs of BLM25 homozygotes and BLM29, 63.1 (±6.8) and 59.1 (±5.6), also differed significantly. BLM29 homozygotes of *qDCI-6* had earlier DCI than BLM25 homozygotes.

### Identification of candidate genes

**Figure 2:**
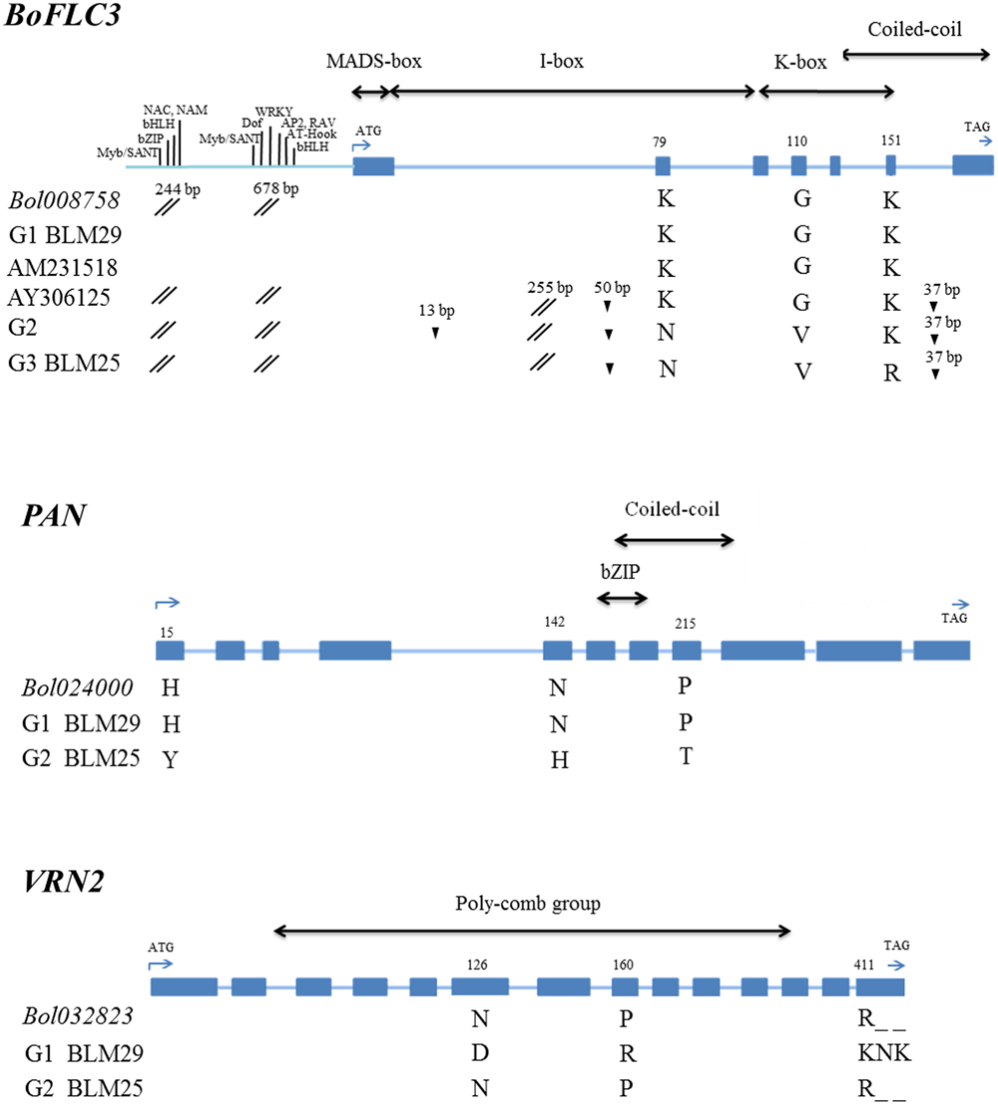
Schematic structures of three candidate genes for DCI and CQ. *BoFLC3* is the most likely candidate gene for *qDCI-3/qCQ-3*, and *PAN* and *VRN2* are the candidate genes for *qDCI-6/qCQ-6*. The sequences of loci *Bol008758*, *Bol02400*, and *Bol032823* corresponding to *BoFLC3*, *PAN*, and *VRN2*, respectively were retrieved from the BolBase database. AM231518 and AY306125 are two different alleles of *BoFLC3* from Chinese kale and cabbage, respectively. Gene structure with the important domains of these three genes are indicated. SNPs causing non-synonymous amino acids are indicated. G1, G2, and G3 are the haplotypes identified from 112 broccoli breeding lines according to the sequence variation. The exons and introns are represented by solid boxes and lines, respectively. The solid triangle (▲), double slash (//), and solid circle (●) indicate insertion, deletion, and pre-mature stop codon, respectively. *BoFLC3* is comprised of 7 exons and 6 introns; and variations of insertion, deletion, and substitution were found in introns 1 and 6; and exons 2, 4, and 6. *PAN* is comprised of 10 exons and 9 introns for which nonsynonymous substitutions were detected on exons 1 and 8 and one early stop codon on exon 10. *VRN2* is comprised of 14 exons and 13 introns, two nonsynonymous substitutions were found on exons 6 and exon 8 and one nonsynonymous substitution and two indel amino acids were detected on exon14. The *cis*-elements on the deletion fragments of *BoFLC3* upstream were predicted by PlantPAN 2.0 using *Arabidopsis* database^46^.

The two regions containing QTL pairs each contained eight genes with predicted functions related to flowering time and floral organ differentiation (Supplementary Table 2), a subset of which showed striking functional differences between the parents after sequence alignment analyses of resequencing data of the candidate genes. In the At5g7910-CHT20 region containing *qDCI-3* and *qCQ-3*, only *Bol008758*, annotated as *FLOWERING LOCUS C* (*FLC*), showed allelic variation between BLM29 and BLM25 (Fig. 2). BLM29 had the same gene sequence as the *B. oleracea* var. *capitata* reference genome but had 244- and 678-bp indels upstream^14^; while 3 SNPs caused non-synonymous substitutions in BLM25 (Fig. 2). *Bol008758* corresponds to *BoFLC3* according to the linkage maps and functions of *FLC* paralogs in *B. oleracea*^13,31^. The *BoFLC3* sequences of BLM29 and BLM25 were identical to those of *B. oleracea* var. *alboglabra* (Chinese kale, AM231518) and var. *capitata* (cabbage, AY306125).

In the fito23-fito36 interval containing *qDCI-6* and *qCQ-6*, the BLM29 allele of *PAN* (*PERIANTHIA*, *Bol024000*), a bZIP-transcription factor required for *AGAMOUS* activation in *Arabidopsis* flowers^32^, has the same amino acid sequence as *Bol024000*; BLM25 has three non-synonymous substitutions (Fig. 2). The BLM25 allele of *VRN2* (*VERNALIZATION2*, *Bol032823*), a vernalization-responsive repressor of *FLC*, was identical to *Bol032823*, cabbage *VRN2*; BLM29 had two non-synonymous substitutions and two additional amino acids on exon 14. The other six candidate genes lacked non-synonymous substitution.

**Figure 3:**
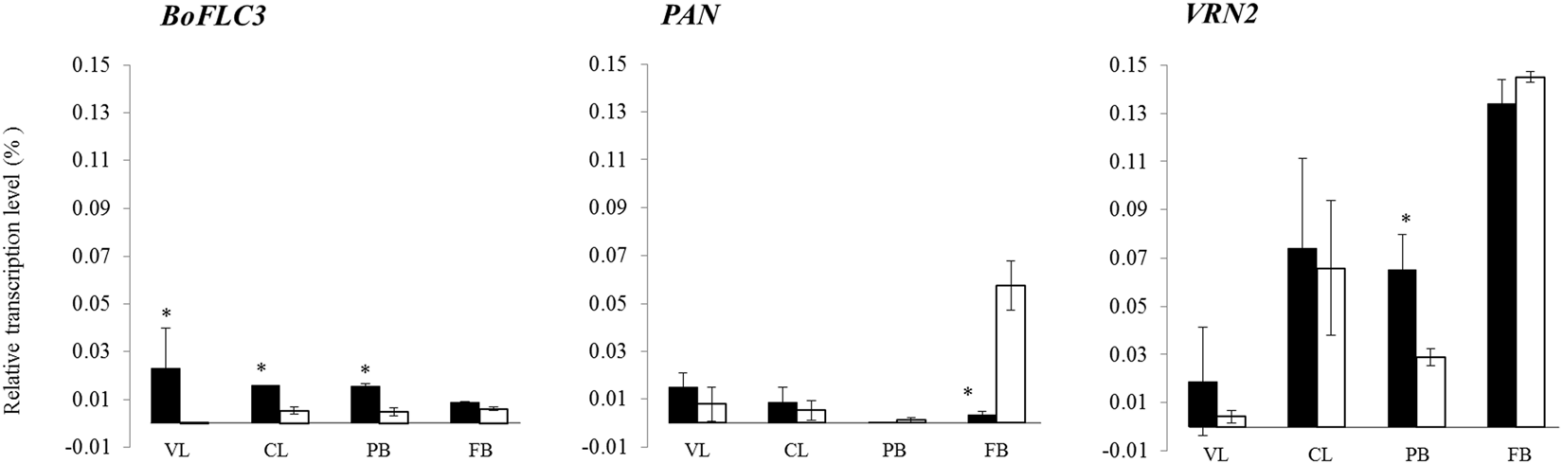
Relative expression of three candidate genes for DCI and CQ. The expression of candidate genes residing in the identified QTL intervals, *BoFLC3*, *PAN*, and *VRN2* were analyzed in four different tissues, the vegetative leaf at 4-leaf stage (VL), the latest leaf at curd forming stage (CL), the peduncle bract at harvest stage (PB), and floral bud at harvest stage (FB). Asterisks indicate significant difference at *p* < 0.05 between the two parents, BLM29 (closed box ■) and BLM25 (open box □), as analyzed by *t* test.

BLM29 and BLM25 showed significantly different expression levels of *BoFLC3* in the vegetative leaf at the 4-leaf stage (VL); the latest leaf at curd forming (CL); and the peduncle bract at harvest stage (PB); *PAN* in floral bud at harvest stage (FB: Fig. 3); and *VRN2* in PB. *BoFLC3* and *PAN* expression levels were relatively low–*VRN2* had the highest expression levels and was clearly not repressed in the subtropical environment.

### Association analysis of candidate genes for days to curd induction

To association between *BoFLC3*, *PAN*, and *VRN2* and DCI, a panel of 95 broccoli-derived and 17 Chinese kale-derived breeding lines, were subjected to DCI measurement and haplotype analysis. After sequencing of the three genes in the 112 breeding lines, 3, 2, and 2 major haplotypes were identified for *BoFLC3*, *PAN*, and *VRN2* (Fig. 2; Supplementary Fig. 4). Haplotypes of *BoFLC3* and *PAN*, but not *VRN2*, accounted for DCI variation revealed by association analysis (Table 2). Among three major haplotypes of *BoFLC3* (Fig. 2; Supplementary Fig. 4a), the DCI of haplotype G1 was significantly earlier than those of G2 and G3 in both 2014 and 2015 (Table 2) with high heritabilities (*H*^*2*^_*B*_ = 0.9 in 2014 and 0.94 in 2015). Haplotype G1 shared the same sequence with the early parent line BLM29. Haplotype G2 exhibited a 13-bp insertion, a 255-bp deletion, and a 50-bp insertion at the first intron, a 37-bp insertion in intron 6, and 1 SNP at exons 2 and 4 leading to non-synonymous substitutions, K79N in an I-box domain and G110V in a K-box domain, respectively. Haplotype G3 shared nearly identical *BoFLC3* sequence with G2 and the late parent, BLM25 except for a 13-bp insertion in intron 1 and a nonsynonymous substitution K151R on exon 6. The DCI of haplotype G1 was significantly earlier than those of G2 and G3 in both 2014 and 2015, indicating that the insertion and deletion on intron 1 and/or nonsynonymous substitutions of *BoFLC3* altered DCI. Two major *PAN* haplotypes, G1 and G2, shared highly similar sequences with BLM29 and BLM25, respectively (Fig. 2, Supplementary Fig. 4b). The two *PAN* haplotypes, with high heritabilities (0.95 in 2014, 0.97 in 2015), had significant differences in average DCI in both years–G1 and G2 were 39.8 (±16.6) and 51.8 (±9.3) days in 2014, and 48.9 (±8.5) and 61.1 (±7.0) in 2015 (Table 2). Two *VRN2* haplotypes shared high similarity with BLM29 and BLM25 (Fig. 2, Supplementary Fig. 4c), respectively, with low heritabilities (0.09 in 2014, 0.19 in 2015). The average DCI for haplotype G1 and G2 were 50.8 (±11.2) and 49.8 (±11.7) days in 2014, and 58.6 (±7.8) and 59.4 (±8.3) days in 2015 (Table 2), non-significant differences between haplotypes.

**Table 2:** The days to curd induction of haplotypes of *BoFLC3, VRN2* and *PAN* in 2014 and 2015.

Gene	Haplotype	No. of individuals	Range (days)		Average (days)^a^	
			2014	2015	2014	2015
*BoFLC3*	G1	17	16–71	38–62	39.8 (±16.6)^b^	48.9 (±8.5)^b^
	G2	17	35–65	49–69	50.8 (±9.5)^a^	59.3 (±5.7)^a^
	G3	78	32–76	49–75	52.1 (±9.3)^a^	61.4 (±7.2)^a^
*PAN*	G1	17	16–71	38–63	39.8 (±16.6)^b^	48.9 (±8.5)^b^
	G2	95	32–76	49–75	51.8 (±9.3)^a^	61.1 (±7.0)^a^
*VRN2*	G1	25	31–66	38–75	50.8 (±11.2)^a^	58.6 (±7.8)^a^
	G2	87	16–76	38–72	49.8 (±11.7)^a^	59.4 (±8.3)^a^

^a^Averages with different superscript letters indicate significant difference at *p* < 0.05 by LSD analy.

Albeit on different chromosomes, strong association was found between *BoFLC3* and *PAN* haplotypes. Only 3 of 6 possible combinations of the three *BoFLC3* and two *PAN* haplotypes occurred in the 112 accessions, *BoFLC3*-_G1_/*PAN*-_G1_, *BoFLC3*-_G2_/*PAN*-_G2_, and *BoFLC3*-_G3_/*PAN*-_G2_ (Table 3), deviating significantly from random association (χ^2^ = 111.2, *p* ≈ 0.001). The average DCIs of *BoFLC3*-_G1_/*PAN*-_G1_ were significantly earlier than *BoFLC3*-_G2_/*PAN*-_G2_, and *BoFLC3*-_G3_/*PAN*-_G2_, which were not significantly different from one another. No breeding lines carried early alleles at one locus and late alleles at the other, e.g., *BoFLC3-*_*G1*_/*PAN-*_*G2*_, *BoFLC3-*_*G2*_/*PAN-*_*G2*_ or *BoFLC3-*_*G3*_/*PAN-*_*G2*_ genotypes, indicating selection against such genotypes in high ambient temperature. Occurrence of the 6 possible *BoFLC3*/*VRN2* genotype combinations slightly deviated from the random expectation (χ^2^ = 7.27, *p* ≈ 0.026), and DCI differences among these combinations were small (Table 3).

**Table 3:** Linkage disequilibrium analysis of *BoFLC3* associated with *PAN* and *VRN2*.

Gene	Haplotype	*BoFLC3*								
		G1 (17)^a^			G2 (17)			G3 (78)		
		O^b^	E	DCI^c^	O	E	DCI	O	E	DCI
*PAN*	G1 (17)	17	2.6	39.8 (±16.6)^b^	0	2.6	N.A.	0	11.8	N.A.
				48.9 (±8.5)^b^			N.A.			N.A.
	G2 (95)	0	14.4	N.A.	17	14.4	50.8 (±9.5)^a^	78	66.2	52.2 (±9.4)^a^
				N.A.			59.3 (±5.7)^a^			61.4 (±7.2)^a^
*VRN2*	G1 (25)	6	3.8	46.0 (±15.8)^a^	7	3.8	47.6 (±9.7)^a^	12	17.4	55.0 (±8.3)^a^
				48.0 (±6.9)^b,c^			57.5 (±4.7)^a,b^			63.0 (±5.4)^a^
	G2 (87)	11	13.2	36.5 (±16.8)^b^	10	13.2	53.1 (±9.1)^a^	66	60.6	51.5 (±9.4)^a^
				49.2 (±9.5)^a,b^			60.4 (±6.1)^a^			61.2 (±8.1)^a^

^a^The number in the bracket () is the total number of breeding lines categorized in each haplotype.

^b^O and E represent the observed and expected breeding lines in each haplotype combination.

^c^The DCIs in the upper and lower rows are the average (±SD) estimated in 2014 and 2015, respectively.

^d^The averages with different superscript letters are significantly different by Fisher’s least significant difference (LSD) test at *p* < 0.05.

## Discussion

Since the Brassicaceae originated in temperate zones, most flowering time mechanisms investigated to date regard long-day photoperiod and vernalization. In this study, 112 advanced broccoli breeding lines selected in subtropical regions provide a unique genetic resource to uncover new genes/QTLs. By bi-parental mapping, 4 DCI QTLs and 3 CQ QTLs accounted for totals of 56.28% and 33.02% of variance in curding time and curd quality (Table 1; Fig. 1). The location of *qDCI-3/qCQ-3* corresponded to *BoFLC3*^13^, and three *BoFLC3* haplotypes were identified in these advanced breeding lines of broccoli (Fig. 2; Supplementary Fig. 4a). Two QTLs, *qDCI-4* and *qCQ-2* might coincide with QTLs of curd induction time uncovered in 111 cauliflower lines cultivated under cool circumstances but not high ambient temperature^26^. The other 2 QTLs, *qDCI-6/qCQ-6* and *qDCI-7* were newly uncovered in these two non-vernalized broccoli lines. Thus, we discovered novel identified genes/QTLs of DCI and CQ with new roles in floral regulation in relatively high ambient temperature under subtropical short-day conditions.

Vernalization, a long duration of low temperature, is a decisive environmental cue triggering flowering in *Arabidopsis* and some crops. *B. oleracea* crops can be classified by vernalization type as biennials (e. g. cabbage, kohlrabi) with non-vernalization types being annuals (e. g. broccoli and cauliflower)^12^. On the other hand, other reports indicate most broccoli cultivars require vernalization with a long duration below 23 °C^4^, but several commercial hybrid and breeding lines form curd at temperatures of 25–32 °C^28,29^. The present results indicated that DCI may be controlled by both vernalization and non-vernalization pathways.

**Figure 4:**
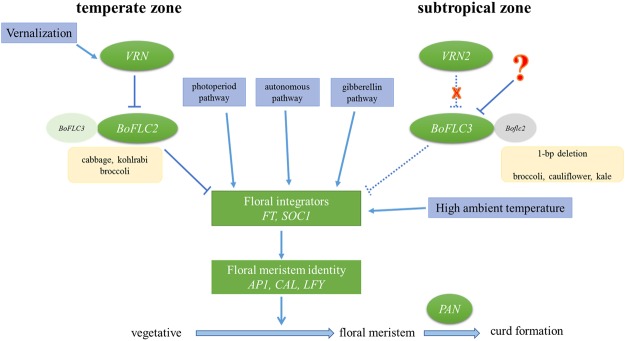
A model for genetic and environmental control of flowering time and curd formation in temperate versus tropical *B. oleracea*. The crops, flowering pathways, and albeit genes are indicated by yellow, blue, and green, respectively.

Duplicated *BoFLC* genes permitted evolution of alternative pathways for control of flowering in temperate and tropical environments (Fig. 4). Gene redundancy contributes to diverse flowering time responses in *Brassica*^15–17^, however no reports to date reveal which of the four recognized *FLC* paralog(s) (*BoFLC1*, *BoFLC2*, *BoFLC3*, and *BoFLC5*) regulate flowering time in non-vernalizing types^12,13,33^. *BoFLC2* explains large FTi variation in the F_2_ progeny of a broccoli (non-vernalization type)× cabbage (vernalization type) cross^12^, with expression reduced dramatically after vernalization, resulting in increased apex *FT* expression^11,23^. Null

*Boflc2* alleles caused by a single base deletion in exon 4 are absent from biennials such as Brussels sprouts and cabbage but widespread in non-vernalizing broccoli, cauliflower, and a rapid cycling line^12,13,34^, including both parental lines BLM29 and BLM25 (Supplementary Fig. 5). It is possible that null function alleles of *Boflc2* have been fixed in the annual *Brassica* crops, such as broccoli, cauliflower, and kale, since vernalization is unfavorable for cultivation of these crops in high temperature areas. In the DH lines of a rapid cycling line (var. *alboglabra*, non vernalization type) × a broccoli line (var. *italica*, non vernalization type), none of the 4 *BoFLC* were associated with FTi by linkage analysis, and *BoFLC4* (*BoFLC2*) and *BoFLC5* did not contribute to flowering time because of premature stop codons^13^. To date, no reports reveal which *FLC* paralog(s) play important role(s) in regulating flowering time in non-vernalization type plants.

*BoFLC3*, rather than *BoFLC2*, was the primary determinant of DCI variation in subtropical broccoli. We found no DCI QTLs near *BoFLC2*, but *qDCI-3* and *qCQ-3* overlapped one another and other flowering QTLs^13,35^, with *BoFLC3* near the likelihood peak showing allelic variation between the parental lines. BLM29 and BLM25 shared the same amino acid sequences as the early flowering line A12DHd which is a rapid-cycling line derived from *B. oleracea* var. *alboglabra* and the late flowering line GDDH33 which is double haploid line derived from F_1_ hybrid Calabrese variety, Green Duke (*B. oleracea* var. *italica*)^13^, respectively. Both BLM29 and A12DHd alleles of *BoFLC3* delayed FTi in the F_2_ and BC_1_S_1_ populations^13^, respectively (Table 1, Fig. 2). *FLC2* dominated flowering time variation in *B. oleracea* and *B. rapa* populations responsive to vernalization, with no QTLs detected near *FLC3*^12,15^. Moreover, *BoFLC3* expressed consistently in cotyledons, leaves, and apex of broccoli and leaves of cauliflower and cabbage^11,12^. Thus, functional variation in *BoFLC3* had little if any phenotypic effect under temperate environments. BLM29 had higher expression of *BoFLC3* than BLM25 in VL, CL, and FL, which might cause earliness (Fig. 3). Thus, allelic effects and differential expression of *BoFLC3* bring out various flowering times in natural broccoli germplasm. How *BoFLC3* inhibits FTi by repressing *FT* and *SOC1* remains of interest and its function is still to be determined.

The expression of *FLC* regulated by vernalization, especially the histone 3 modification on intron 1 by the activities of both VRN1 and VRN2, is well known and extensively investigated^36^. *VRN2*, corresponding to *Bol032823*, was a positional candidate gene for the major QTL on chromosome O6, *qDCI-6/qCQ-6*. *VRN2* showed allelic variation in BLM29, BLM25, and cabbage (Fig. 2). Under the subtropical environment, expression levels of *VRN2* were neither repressed nor significantly different between two parental lines (Fig. 3). Furthermore, genetic association between *BoFLC3* and *VRN2* was relatively small, a finding which might be caused by *VRN2* being closely linked to the other real flowering gene(s) (Table 3). Together with lack of significantly different DCI between haplotypes, the results herein did not support that *VRN2* regulated *BoFLC3* to cause DCI variation in subtropical germplasm. *BoVRN2*/*BoFLC2*-independent mechanisms were suggested in temperature-regulated floral transition in cauliflower because of a lack of consistent relevance between gene functions of *BoVRN2* and *BoFLC2*^26^, and *FLC*-independent vernalization pathways were also suggested in broccoli and *Arabidopsis*^37^. Non-vernalization pathways are suggested to regulate DCI in subtropical germplasm, leading from obligate to facultative vernalization flowering.

Improved thermal tolerance of broccoli, reducing unfavorable characters such as leafy curds and uneven-sized flower buds^3^, was associated with *qDCI-6/qCQ-6* here and a previously-published QTL^22^, for which *PAN* is the most likely candidate (Table 2; Fig. 2). The highest gene expression of *PAN*, a gene in which mutation changed shoot apical meristem size and floral organ number^32^, was detected in the FB of BLM25 (Fig. 3), perhaps contributing to good curd quality. One QTL on chromosome O6 induced curd formation when cauliflower was cultivated under temperatures >22 °C^22^. *BoAP1-a* was strongly suggested to control curd induction and bract development^22,38^; however, no amino acid variation was detected between BLM29 and BLM25 – indeed, only one SNP occurred even −139 bp upstream of *BoAP1*. Nevertheless, further investigations are needed to prove the role of *PAN*.

The wide range of flowering times in natural population is regulated not only by multiple genes in accordance to environmental cues but also by intrinsic non-linear gene interactions (epistasis). The most well-known of these interactions is that *FLC* gene expression is repressed by VRN2 and VIN3, and consequently the expression of *FT* and *SOC* is activated^10,36^. Digenic interaction contributed 3.2% heat tolerance variation in a broccoli DH population^39^. In the BLM29 × BLM25 F_2_ population, two digenic interactions accounted for DCI but not CQ variation, in which *qDCI-6* interacted with *qDCI-3* and *qDCI-7*, respectively (Table 1). Thus, the novel QTL, *qDCI-6*, played an important role in regulating FTi by interacting with other genes. The candidate gene for *qDCI-6*, *PAN*, was strongly associated with *BoFLC3* in 112 subtropical broccoli breeding lines (Table 3), strongly suggesting that specific combinations of *BoFLC3* and *PAN* haplotypes confer selective advantages under relatively high ambient temperature and short-day length.

The adjustment of crop flowering time has been a breeding goal of widespread importance to maximize harvestable yields and shift cultivation seasons to meet market demands. To promote broccoli to form curd too early in Southeast Asia would risk heat stress, resulting in various-sized floral buds, poor curd quality with several bracts of leaves and perhaps even failure of curd initiation (Fig. 1a). Alleles conferring earliness and good curd quality are both necessary to commercially successful elite cultivars. In this study, BLM29 provided the favorable allele, promoting curding by 3.81 days and reducing curd leafiness by 0.44 degree, from *qDCI-6/qCQ-6* (Table 1). After 3 backcross generations with marker-assisted selection, four selected BC_3_F_1_ individuals exhibited earlier flowering time than the elite parent line BLM25 but possesses similarly superior horticultural traits (Supplementary Figure 3). In this study, the novel FTi QTL, *qDCI-6/qCQ-6* conferring curd induction time and curd quality provides a valuable genetic resource for breeding broccoli, cauliflower and other Brassica vegetable cultivars optimized for yield and quality in the climatic zones of relatively high ambient temperature.

Evolution of alternative pathways for control of flowering in temperate and tropical environments respectively, based on duplicated genes, might be utilized in adaptation of numerous taxa to new climates, either by searching for naturally occurring gene duplicates or using gene engineering and/or editing approaches. As ambient temperatures rise, plant adaptation to diverse geography and maximum crop production is likely to involve extensive modification of temperature responses, as exemplified by the adaptation of *B. oleracea* to Taiwan. Indeed, reconciling current models for the genetics of flowering with results from divergent latitudes or warmer climates requires new dimensions (Fig. 4). Linearity and additivity may be inadequate in such models – the wide range of flowering times in natural populations is regulated not only by individual gene effects but also by non-linear interactions (epistasis) such as *qDCI-6* with *qDCI-3* and *qDCI-7*, respectively (Table 1). This study provided new insight into FTi regulation and implemented a strategy to breed new elite lines with earliness, demonstrating an example of translational agriculture.

## Methods

### Plant material

To identify QTLs conferring curd induction time and curd quality in broccoli, *B. oleracea* var. *italica*, an F_2_ population derived from a cross between two commercial breeding lines, BLM25 and BLM29, was established. BLM25, with superior curd quality and horticultural characters but with late curding time was used as a female parent in the F_1_ cross and as the recurrent parent in subsequent marker-assisted backcrossing. BLM29, a kale-derived broccoli breeding line with early curd formation but inferior curd quality was the male parent and donor parent. The randomly selected 94 and 188 F_2_ individuals were used to construct a primary linkage map and map QTLs conferring two important traits, days to induction (DCI) and curd quality (CQ). The 188 F_2_ individuals were cultivated in conventional cool seasons starting from Oct 1, 2010 to Jan 14, 2011 to evaluate DCI, and their derived F_2.3_ families were grown in the hot season starting from July 12 and initiated curd formation about September 10, 2011 to evaluate CQ.

A panel of 112 advanced broccoli breeding lines were genotyped to identify haplotypes of candidate genes for association genetic analysis, and phenotyped in the fall-winter of 2014 and 2015. All plants were grown at the experimental station of Known-You Seed Co., Ltd, Xinshi, Tainan, Taiwan (23.079337°N, 120.295377°E).

### Assessment of days to induction and curd quality

Days to induction (DCI) for each F_2_ individual was determined by the duration from the day of seedling transplantation to the field until curd initiation, indicated by visible curd of approximately 0.5 cm diameter. Curd quality (CQ) of each F_2_ individual was estimated by the arithmetic mean of 30 F_2:3_ individuals. CQ, reflected by curd shape, was evaluated based on curd leafiness reflected by abundance of bracts appearing between inflorescence internodes at harvest. CQ index was scored from 1 to 4 according to the abundance of bracts, from none, few, several, to numerous bracts (Fig. 1).

### Genotyping assay of molecular markers

Genomic DNA extraction and genotype assays of PCR-based markers were as described previously^40,41^. SSR markers with prefixes of BnGMS, Ol, BoGMS, fito, BRMS, Na, CB, BRAS, SORA, and BrGMS were retrieved from previous publications and described well in Shuang *et al*.^40^ and intron polymorphic (IP) markers with prefix At were adopted from Panjabi *et al*.^42^. After genotype assay, 117 of 402 SSR markers and 61 of 100 IP markers exhibited polymorphism between BLM25 and BLM29. To fill linkage gaps, SSRs with prefix of CHT were mined from a whole genome shotgun sequence of BLM29 obtained by 454 (Roche), and the primer information was listed as in Supplementary Table 4.

### Genetic mapping of QTLs conferring curd induction time and curd quality

The primary linkage map was constructed by using 94 randomly selected F_2_ progeny genotyped with 126 polymorphic markers, including 107 SSR and 19 IP markers distributed on the 9 broccoli chromosomes (Supplementary Figure 1). For interval mapping of QTLs, a total of 188 F_2_ individuals were genotyped with 60 markers at average spacing of approximately 15 cM. The linkage map was constructed by using MapMaker Exp 3.0 with a LOD threshold of 3.5^43^. To identify QTLs of DCI and CQ, a multiple-QTL mapping analysis based on Haley-Knott regression was performed using R/qtl^44^. Forward QTL mapping analysis was applied, with a single-QTL genome scan performed first and followed with incorporation of QTL with the largest effect into the model by functions *scanone* and *makeqtl*. Additional additive QTL and interaction pairs were then scanned and evaluated in the model by functions *scantwo*, *makeqtl*, and *addint*. The map position of each QTL in the model was refined by function *refineqtl*. The whole procedure was repeated until no more significant QTL was detected.

### Breeding elite early curding lines by marker-assisted selection

The scheme for breeding elite early curding lines by marker-assisted selection (MAS) was illustrated on Supplementary Figure 2. The 188 F_2_ individuals used for QTL analysis were self-pollinated to generate 188 F_2:3_ families which were genotyped with fito203 and fito036, flanking *qDCI-6*, for foreground selection and 22 markers for background selection. Three F_2:3_ were selected and backcrossed to the elite parent, BLM25, to generate the BC_1_F_1_ population. After MAS with 50 markers, 10 of 300 BC_1_F_1_ individuals were subsequently backcrossed to BLM25 to generate 192 BC_2_F_1_ individuals. After MAS, 4 selected BC_2_F_1_ individuals were self-pollinated several times to generate 128 BC_2_F_2_ individuals and backcrossed to BLM25 to generate 128 BC_3_F_1_ individuals. Four of 128 BC_3_F_1_ individuals were selected and were consequently self-pollinated several times to generate 90 BC_3_F_2_ individuals.

Only 113 BC_2_F_2_ and 87 BC_3_F_2_ individuals were grown from February 15 and October 5, 2015 respectively, to evaluate DCI and genotyped with fito203 and fito036 to predict the genotypes of *qDCI-6*. Statistical summary parameters, one-way analysis of variance, and Fisher’s least significant difference (LSD) were calculated by using R^45^.

### Identification of candidate genes for curd induction and curd quality

The whole genome sequence of *B. oleracea*, assembly GCA_000695525.1, was obtained from EnsemblPlants (http://plants.ensembl. org/index.html). Gene annotation was performed for 2 major QTL intervals, At5g07910/CHT20 on chromosome O3 and fito203/fito036 on chromosome O6. Genes with putative functions in flowering pathways and floral organ differentiation were considered candidates for DCI and CQ.

Sequences of candidate genes were downloaded from a *Brassica* database (http://brassicadb.org/). Candidate genes were sequenced from approximately 1,000 bp upstream of the transcription start site to 100–300 bp downstream of the transcription stop codon. In addition to sequencing the candidate genes residing in the two major QTL intervals, one important flowering time gene, *BoFLC2*, was genotyped according to Ridge *et al*.^34^.

### Quantitative real-time PCR analysis of the expression of candidate genes

RNA from BLM29 and BLM25 was extracted from approximately 1 g of four tissues at the vegetative leaf at 4-leaf stage (VL), the latest leaf at curd forming stage (CL), the peduncle bract at harvest stage (PB), and floral bud at harvest stage (FB) by using TRIzol Reagent (Invitrogen, USA). The first-strand cDNA was synthesized with 1 μg total RNA using PrimeScript™ RT Reagent Kit (Perfect Real Time, TAKARA Bio Inc., Japan) in a volume of 10 μL, and qPCR was carried out using an ABI 7500 Sequence Detection System (ABIPRISM; Applied Biosystems, USA) with the KAPA SYBR^®^ FAST qPCR Kits (KAPA Biosystems, USA) in a total volume of 20 μL. The relative expression of genes was calculated by using *BoActin* as the internal control.

### Association analysis of DCI candidate genes

To identify the most likely DCI candidate genes, DCI of 112 commercial broccoli breeding lines were estimated and candidate genes sequenced. The broccoli was grown in Xinshi Dist., Tainan, in the conventional cool seasons starting from September 2014 and 2015, respectively. DCI of each line was estimated from the average of 8–10 individuals. Broad sense heritability was calculated as mean square of genotype divided by total mean square (*H*^*2*^_*B*_ = V_G_/V_P_). The haplotypes of breeding lines were identified according to major DNA variants of the candidate genes.

## Electronic supplementary material

Supplementary Dataset 1
