# Supplementary material for: Subtropical adaptation of a temperate plant (Brassica oleracea var. italica) utilizes non-vernalization-responsive QTLs
Source: Sci Rep. 2018 Sep 11;8:13609. doi: 10.1038/s41598-018-31987-1 (PMC6134136; doi:10.1038/s41598-018-31987-1)
Supplement: Supplementary file 1 — Supplementary Dataset 1 [file 41598_2018_31987_MOESM1_ESM.pdf]

Subtropical adaptation of a temperate plant (*Brassica oleracea* var. *italica*) utilizes non-vernalization-responsive QTLs

Yann-rong Lin[1], Jou-yi Lee[1, 2], Meng-chun Tseng[1], Chieh-ying Lee[3], Chian-he Shen[3], Chun-shan Wang[1], Chia-ching Liou[1], Lan-shuan Shuang[1, 4], Andrew H. Paterson[4], Kae-kang Hwu[1,*]

[1] Department of Agronomy, National Taiwan University, Taipei 10617, Taiwan.

[2] Department of Horticulture, Chiayi Agricultural Experiment Station, Taiwan Agricultural Research Institute, Chiayi 60044, Taiwan

[3] Known-You Seed Co., LIR, Kaohsiung 84043, Taiwan.

[4] Plant Genome Mapping Laboratory, University of Georgia, Athens, Georgia 30602, USA.

***Corresponding author**

Kae-kang Hwu

Department of Agronomy, National Taiwan University

No. 1, Sec. 4, Roosevelt Road, Taipei 10617, Taiwan

TEL: 886-2-3366-4761; FAX: 886-2-2362-0879

E-mail: khwu@ntu.edu.tw

Supplementary Table 1 The genotypic effects of *qDCI-6* on days to curd induction in BC_2F_2 and BC_3F_2 cultivated in 2014 and 2015 respectively.

Population / *qDCI-6* genotype	BC_2F_2[a]			BC_3F_2[a]		
	No. of individuals	Range	Average[b]	No. of individuals	Range	Average[b]
BLM 25 homozygotes	13	38-53	48.9 (±4.6)^{a}	15	57-70	63.1 (±6.8)^{a}
Heterozygotes	81	34-53	42.3 (±5.2)^{b}	61	52-70	61.4 (±3.8)^{ab}
BLM 29 homozygotes	19	34-53	39.8 (±5.2)^{b}	11	52-66	59.1 (±5.6)^{b}

[a] The populations of BC_2F_2 and BC_3F_2 were grown in 2014 and 2015, respectively.

[b] The averages with different superscript letters are significantly different by Fisher's least significant difference (LSD) test at $p < 0.05$.

Supplementary Table 2 The list of candidate genes for DCI and CQ

Locus	Physical position		Annotated gene	Predicted function
	Starting	Ending		
qDCI-3/qCQ-3				
At5g7901	1496431			
Bol008816	1597880	1598473	*ISOPRENYL CYSTEINE METHYLTRANSFERASE B* (*ICMTB*)	Isoprenyl cysteine carboxyl methyl transferase (ICMT) family; flower development
Bol008813	1609908	1607633	*ALPHA-GALACTOSIDASE 2* (*AGAL2*)	Alpha-galactosidase activity, postive regulation of flower development, leaf morphogenesis
Bol008811	1616803	1621522	*TRANSDUCIN/WD40 REPEAT-LIKE SUPERFAMILY PROTEIN* (*WD40 REPEAT 2*)	Transducin family protein; vegetative to reproductive phase transition of meristem
Bol008807	1635460	1630028	*HISTONE DEACETYLATION COMPLEX 1* (*HDC1*)	Facilitates histone deacetylation. Mutants are hypersensitive to ABA during germination, grow less and flower later than wildtype
Bol008801	1666223	1663830	*WD-40 REPEAT 26* (*WDR26*)	Transducin family protein; vegetative to reproductive phase transition of meristem
Bol008817	1595498	1596205	*TCP DOMAIN PROTEIN 21* (*TCP21*)	TCP family transcription factor; regulation of circadian rhythm
Bol008782	1754459	1757661	*HEAT SHOCK COGNATE* (*HSC70-5*)	Protein folding, response to cadmium ion, response to heat, response to salt stress, responese to virus
Bol008758	1890867	1893743	*FLOWERING LOCUS C* (*FLC*).	K-box region an MDS- box transciption factor family
CHT20	2018894			
qDCI-6/qCQ-6				
fito203	4778308			
Bol032969	5139445	5143284	*SIN3-LIKE 4* (*SNL4*)	RNA polymerase II transcription corepressor activity, histone deacetylase activity
Bol032823	6802971	6805448	*VERNALIZATION2* (*VRN2*)	Maintains *FLC* repression after a cold treatment, serving as a mechanism for the cellular memory of vernalization
Bol032834	6676947	6677735	*FRIGIDA interacting protein 2* (*FIP2*)	Encodes one of the *FRI* interacting proteins : FRIGIDA INTERACTING PROTEIN 1 (FIP1); a major determinant of natural variation in Arabidopsis flowering time
Bol023902	7204783	7203812	*TEOSINTE BRANCHED1/CYCLOIDEA/PCF 15* (*TCP15*)	Circadian rhythm, gynoecium development, inflorescence development, response to cytokinin
Bol023933	7477579	7476604	*NAC DOMAIN CONTAINING PROTEIN* (*NAP*)	Expressed in floral primordia and upregulated by *AP3* and *PI*; associated with leaf senescence, flower develpomet
Bol023961	7705861	7707885	*APETALA1* (*AP1*)	Encodes MADS-box transcription factor protein; specifies floral meristem and sepal identity
Bol024000	8100256	8102971	*PERIANTHIA* (*PAN*)	bZIP transcription factor family protein; is essential for *AG* activation in early flowers of short-day-grown plants
Bol024026	8312095	8313664	*JAGGED* (*JAG*).	C2H2 and C2HC zinc fingers super family; together with *NUB*, it is involved in stamen and carpel development
fito036	8738436			

Supplementary Table 3 The Days to curd induction of haplotypes of *BoFLC3*, *VRN2* and *PAN* in 2014 and 2015

Gene	Haplotype	No. of individuals	Range (days)		Average (days)[a]	
			2014	2015	2014	2015
BoFLC3	G1	17	16-71	38-62	39.8 (±16.6)[b]	48.9 (±8.5)[b]
	G2	17	35-65	49-69	50.8 (±9.5)[a]	59.3 (±5.7)[a]
	G3	78	32-76	49-75	52.1 (±9.3)[a]	61.4 (±7.2)[a]
PAN	G1	17	16-71	38-63	39.8 (±16.6)[b]	48.9 (±8.5)[b]
	G2	95	32-76	49-75	51.8 (±9.3)[a]	61.1 (±7.0)[a]
VRN2	G1	25	31-66	38-75	50.8 (±11.2)[a]	58.6 (±7.8)[a]
	G2	87	16-76	38-72	49.8 (±11.7)[a]	59.4 (±8.3)[a]

[a] Averages with different superscript letters indicate significant difference at $p < 0.05$ by LSD analysis

Supplementary Table 4 Primer combinations used for genetic linkage map of *Brassica oleracea* genome.

Chromosome position	Marker name	Forward primer (5'-3')	Reverse primer (5'-3')	Ref. size	Motif repeat type	Product size	
						BLM25	BLM29
C01	CHT35	ATCATTCAAACAGAGCTTCC	ATTGCCTCTAGTTTCACACG	190	ATC	220	200
	CHT11	TGTAAACACTTTCTCTTTGCC	TTAGGAGAAGTGTGTGACCC	241	ATT	240	200
	CHT106	AGCTCCTTAGTCATGTCTGC	TCAAGGATACGGCTACTAGG	307	TCT	300	312
C02	CHT27	AGTACGAGGAAGCATTTAGC	TTGAAAGAAGTGTTTGTTCG	282	TA	270	260
	CHT59	TCGTCTTGAGGAGTAGAAGG	CCTGAGGACAGAGAGAAGG	321	GGC	332	329/344
	CHT21	GCTTATGGAGTCAACTAGCG	CCGTAATAATCAGAGTTCGC	300	ATG	267	264/276/279
C03	CHT20	ACAAATCTTGGTTTTGAAGC	CAAGACATGAGAAACACGC	186	CTC	140	180
	CHT150	GGATTCGTTGATAACTTTGC	AGTCAAAAGAATCAACACCG	282	AG	285	283
	CHT139	ACCATGATCTGTTCTTGACC	CCTGAGGACAGAGAGAAGG	353	TCT	350	356
C04	CHT53	GATTGTTGTTTATTCGAGGG	AGAGAATCTGACGACAAAGC	225	GAA	200	220
	CHT199	CAAGTACTTCGGTAAATGGC	CAATCTTGAACCAAGAAACC	172	AT	150	160
	CHT3	GCAATGGTGAAAAGTGTAGC	TTCATACTCAGTGATAGCCG	203	AAT	100	200
	CHT4	GATGAATAATATTGCCGACG	TCTCCTTCAAGTCAGAAAGC	109	GAA	83	107
C05	CHT93	TTCAGAATTGAATCAACACG	GCTATGTCGTACTCAGATTCC	237	AG	220	210
	CHT37	TTCGATGTAATCAGTCAACG	GCCCATTAAGTATGTTTTCC	166	AT	160	180
C06	CHT72	AGGTGTTCAGTTCTCATTGG	AATCAGAACATCAAAATCCG	311	AG	315	313
	CHT120	GCACGTAAACCTAAATACGC	CTTCTACCGTCTCTCATTCC	138	AG	140	145
	CHT104	TCTGTCCATAACACACAACC	CCAAATTGACTATTTCTGCC	207	ATA	229	202
C07	CHT6	TACTGTCTATGGTTCGTCCG	CGATTGCTAGAGAGAAGTGG	334	CT	328	298
C08	CHT113	GTTGTTCCGTTTAAGTCAGG	CACAAAAGGAGGAGAGACC	265	GCT	259	265
	CHT33	GATCTGATCAGCTATGCTCC	AAACGCTAGTAATCCTCTCG	161	CCA	180	160
	CHT79	TTCGTAACGTAACCAAGACC	AGTCAAAAGAATCAACACCG	396	TGA	402	396

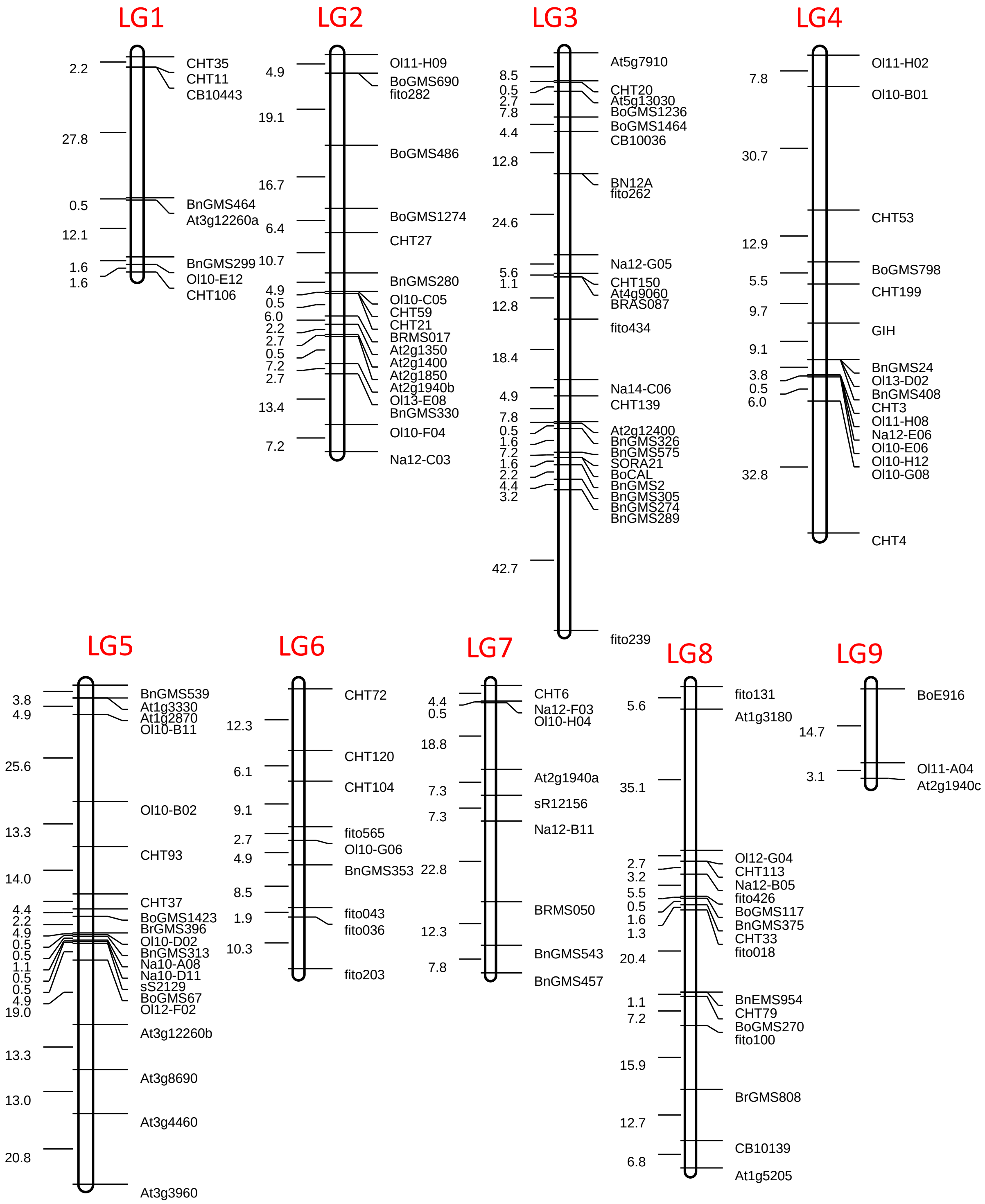

Supplementary Figure 1 Primary genetic linkage map of *B. oleracea* var. *italica*. A primary linkage map was constructed with 94 F_2 of BLM25 × BLM29 genotyped with 126 markers. Nine linkage groups corresponded to the 9 *B. oleracea* chromosomes, spanning 866.6 cM with an average of 7.4 cM between adjacent markers, in a range of 0.5-42.7 cM. The genetic distances on the left between two markers are indicated in centiMorgans (cM), and the names of markers are shown to the right of the linkage groups.

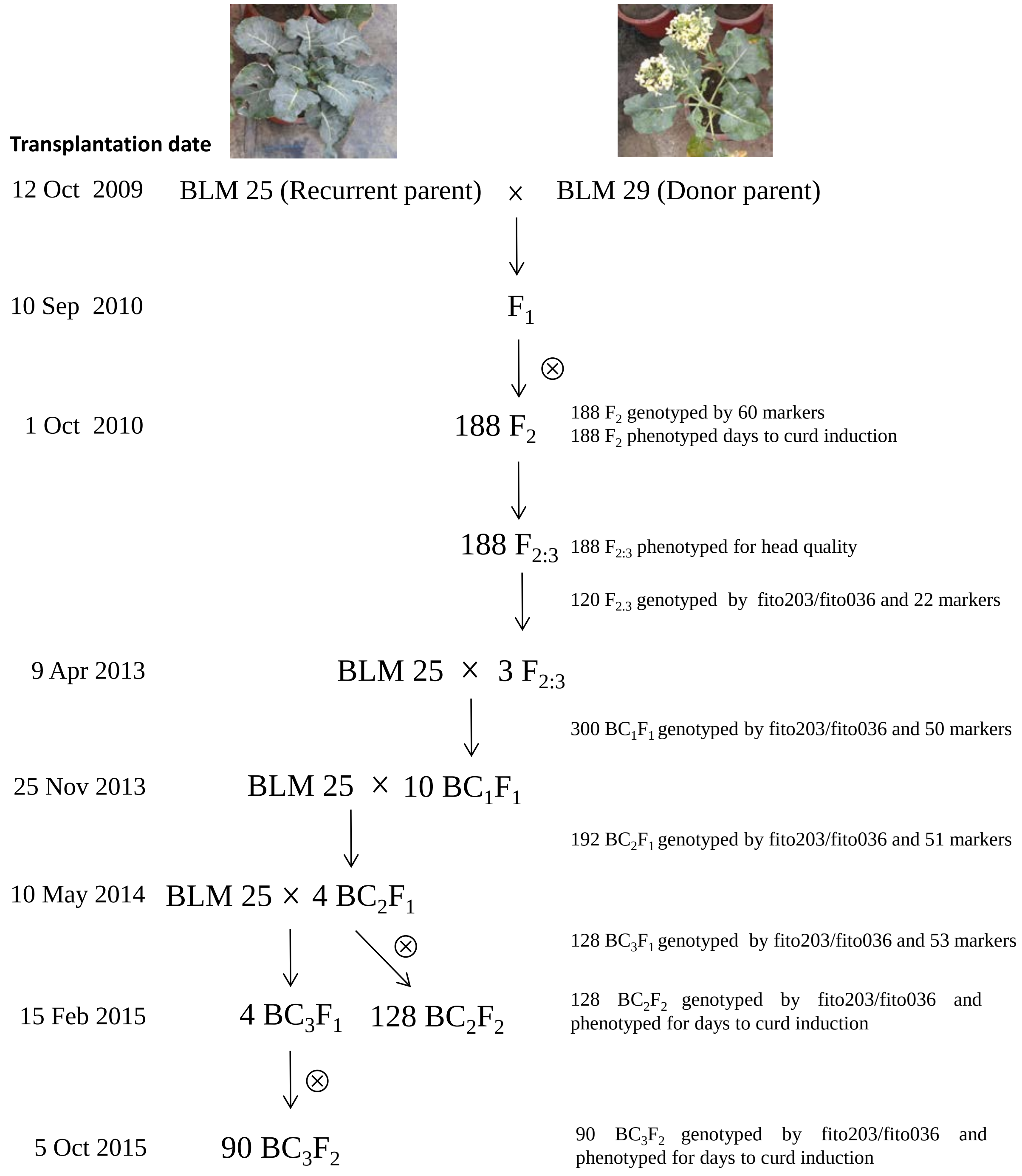

Supplementary Figure 2 QTL mapping of days to curd induction (DCI) and curd quality (CQ) and marker-assisted selection (MAS) for early DCI in broccoli. The 188 F_2 of early breeding line BLM 29 × late breeding line BLM 25 were used for interval mapping of QTLs conferring DCI and CQ. Foreground selection of the $F_{2.3}$ individuals used fito203 and fito036, markers flanking *qDCI-6* and *qCQ-6*. The three selected $F_{2.3}$ were backcrossed to the elite breeding line BLM 25. The BC_2F_2 and BC_3F_2 populations were used to evaluate the effects of identified QTLs.

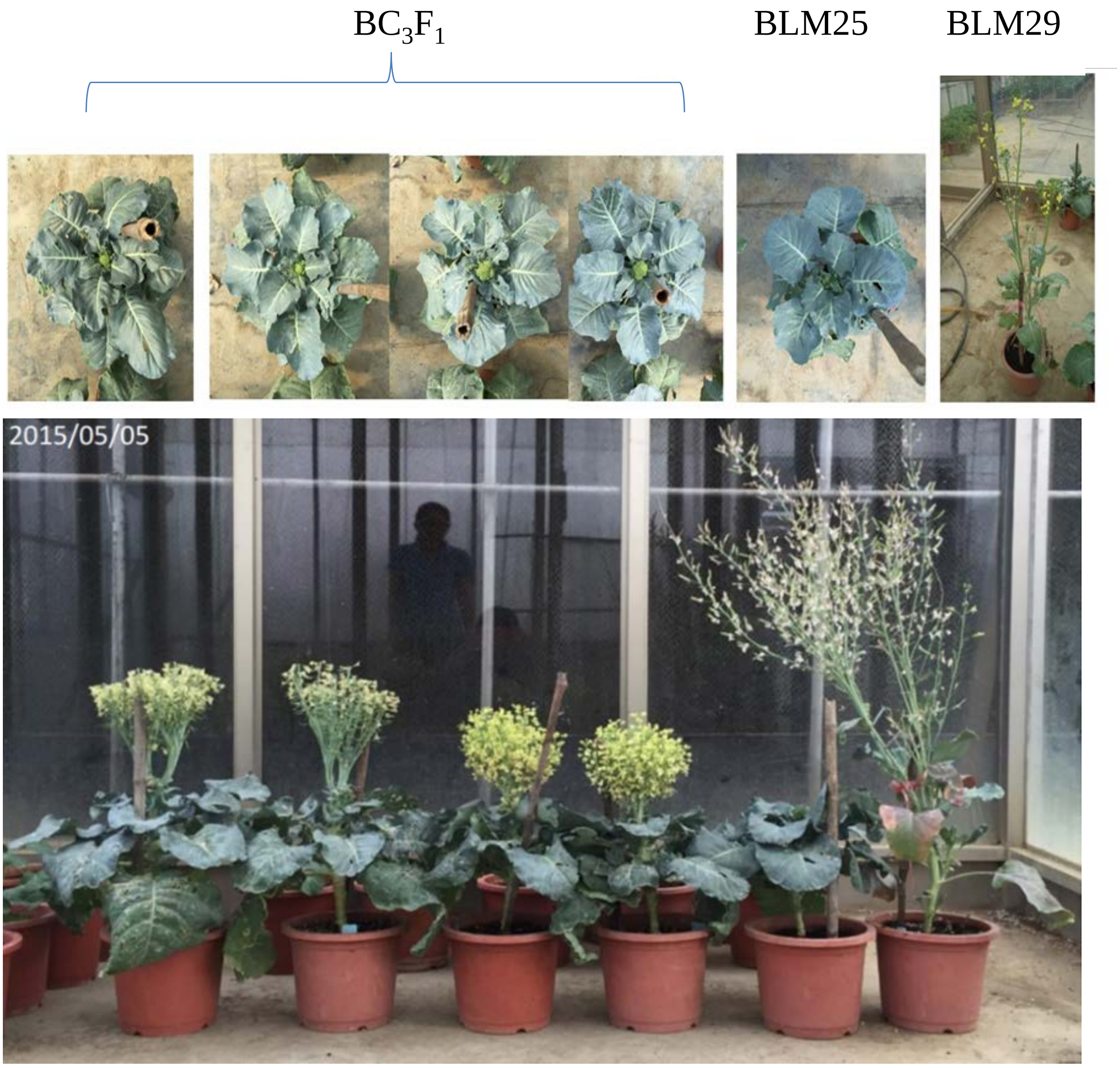

Supplementary Figure 3 Plant morphologies of two parents and selected BC_3F_1 individuals. The photos were shot at curd initiation (above) and flowering of the 4 BC_3F_1 individuals (bottom).

a

BoFLC3	DCI 2014	DCI 2015		234-246	282	284	286	297	298	355	356	357	615	616	649	769	836	871	974	975	976-123	1241	1322	1363	1378	1407	1452-1501	1603	1604	1667	1890-1899	1965	2015	2050	2189	2209-2211	2230-2231	2255	2288	2644	2725	2798	2925	2941	2945-2955	2987-3023	3125	3172	3211	3247-3248	3252
				intron 1																										exon2	intron2				intron3				exon4	intron5	exon6	intron6									
reference				.	A	A	T	-	C	T	T	A	T	A	A	T	C	G	T	T	.	A	C	A	C	T	.	.	.	A	.	C	C	C	A	.	.	A	G	T	A	G	G	T	.	.	T	G	A	.	G
BLM29				.	.	.	.	.	.	.	.	.	.	.	.	.	.	.	.	.	.	.	.	.	.	.	.	.	.	.	.	.	.	.	.	.	.	.	.	.	.	.	.	.	.	.	.	.	.	.	.
GWAS-191	16			.	.	.	.	.	.	.	.	.	.	.	.	.	.	.	.	.	.	.	.	.	.	.	.	.	.	.	.	.	.	.	.	.	.	.	.	.	.	.	.	.	.	.	.	.	.	.	.
GWAS-192	16	48		.	.	.	.	.	.	.	.	.	.	.	.	.	.	.	.	.	.	.	.	.	.	.	.	.	.	.	.	.	.	.	.	.	.	.	.	.	.	.	.	.	.	.	.	.	.	.	.
GWAS-202	26	57		.	.	.	.	.	.	.	.	.	.	.	.	.	.	.	.	.	.	.	.	.	.	.	.	.	.	.	.	.	.	.	.	.	.	.	.	.	.	.	.	.	.	.	.	.	.	.	.
GWAS-195	31	48		.	.	.	.	.	.	.	.	.	.	.	.	.	.	.	.	.	.	.	.	.	.	.	.	.	.	.	.	.	.	.	.	.	.	.	.	.	.	.	.	.	.	.	.	.	.	.	.
GWAS-206	31	54		.	.	.	.	.	.	.	.	.	.	.	.	.	.	.	.	.	.	.	.	.	.	.	.	.	.	.	.	.	.	.	.	.	.	.	.	.	.	.	.	.	.	.	.	.	.	.	.
GWAS-207	31	38		.	T	.	A	.	T	C	-	-	.	.	.	.	.	.	.	.	.	.	.	.	.	.	.	.	.	.	.	.	.	.	.	.	.	.	.	.	.	.	.	.	.	.	.	.	.	.	.
GWAS-200	36	57		.	.	.	.	.	.	.	.	.	.	.	.	.	.	.	.	.	.	.	.	.	A	.	.	G	.	.	.	.	.	T	G	.	.	.	.	A	.	.	.	A	.	.	.	C	T	.	A
GWAS-201	36	38	G1	.	T	.	A	.	T	C	-	-	.	.	.	.	.	.	.	.	.	.	.	.	.	.	.	.	.	.	.	.	.	.	.	.	.	.	.	.	.	.	.	.	.	.	.	.	.	.	.
GWAS-203	36	50		.	.	.	.	.	.	.	.	.	.	.	.	.	.	.	.	.	.	.	.	.	A	.	.	G	.	.	.	.	.	T	G	.	.	.	.	A	.	.	.	A	.	.	.	C	T	.	A
GWAS-204	36	50		.	.	.	.	.	.	.	.	.	.	.	.	.	.	.	.	.	.	.	.	.	.	.	.	.	.	.	.	.	.	.	.	.	.	.	.	.	.	.	.	.	.	.	.	.	.	.	.
GWAS-205	36	57		.	.	.	.	.	.	.	.	.	.	.	.	.	.	.	.	.	.	.	.	.	.	.	.	.	.	.	.	.	.	.	.	.	.	.	.	.	.	.	.	.	.	.	.	.	.	.	.
GWAS-196	41	38		.	.	.	.	.	.	.	.	.	.	.	.	.	.	.	.	.	.	.	.	.	.	.	.	.	.	.	.	.	.	.	.	.	.	.	.	.	.	.	.	.	.	.	.	.	.	.	.
GWAS-197	41	38		.	.	.	.	.	.	.	.	.	.	.	.	.	.	.	.	.	.	.	.	.	.	.	.	.	.	.	.	.	.	.	.	.	.	.	.	.	.	.	.	.	.	.	.	.	.	.	.
GWAS-193	61	62		.	.	.	.	.	.	.	.	.	.	.	.	.	.	.	.	.	.	.	.	.	.	.	.	.	.	.	.	.	.	.	.	.	.	.	.	.	.	.	.	.	.	.	.	.	.	.	.
GWAS-198	66			.	.	.	.	.	.	.	.	.	.	.	.	.	.	.	.	.	.	.	.	.	.	.	.	.	.	.	.	.	.	.	.	.	.	.	.	.	.	.	.	.	.	.	.	.	.	.	.
GWAS-199	66			.	.	.	.	.	.	.	.	.	.	.	.	.	.	.	.	.	.	.	.	.	.	.	.	.	.	.	.	.	.	.	.	.	.	.	.	.	.	.	.	.	.	.	.	.	.	.	.
GWAS-194	71			.	.	.	.	.	.	.	.	.	.	.	.	.	.	.	.	.	.	.	.	.	.	.	.	.	.	.	.	.	.	.	.	.	.	.	.	.	.	.	.	.	.	.	.	.	.	.	.
GWAS-172	35	61		I	.	T	A	A	.	.	.	-	-	-	T	.	G	T	A	G	D	T	G	G	A	G	I	T	T	C	D	A	G	T	G	D	I	C	T	A	.	A	A	A	I	I	G	C	T	D	A
GWAS-168	39	56		I	.	T	A	A	.	.	.	-	-	-	T	.	G	T	A	G	D	T	G	G	A	G	I	T	T	C	D	A	G	T	G	D	I	C	T	A	.	A	A	A	I	I	G	C	T	D	A
GWAS-184	40	61		I	.	T	A	A	.	.	.	-	-	-	T	.	G	T	A	G	D	T	G	G	A	G	I	T	T	C	D	A	G	T	G	D	I	C	T	A	.	A	A	A	I	I	G	C	T	D	A
GWAS-153	43	52		I	.	T	A	A	.	.	.	-	-	-	T	.	G	T	A	G	D	T	G	G	A	G	I	T	T	C	D	A	G	T	G	D	I	C	T	A	.	A	A	A	I	I	G	C	T	D	A
GWAS-122	44	59		I	.	T	A	A	.	.	.	-	-	-	T	.	G	T	A	G	D	T	G	G	A	G	I	T	T	C	D	A	G	T	G	D	I	C	T	A	.	A	A	A	I	I	G	C	T	D	A
GWAS-155	44	56		I	.	T	A	A	.	.	.	-	-	-	T	.	G	T	A	G	D	T	G	G	A	G	I	T	T	C	D	A	G	T	G	D	I	C	T	A	.	A	A	A	I	I	G	C	T	D	A
GWAS-188	44	67		I	.	T	A	A	.	.	.	-	-	-	T	.	G	T	A	G	D	T	G	G	A	G	I	T	T	C	D	A	G	T	G	D	I	C	T	A	.	A	A	A	I	I	G	C	T	D	A
GWAS-106	49	49		I	.	T	A	A	.	.	.	-	-	-	T	.	G	T	A	G	D	T	G	G	A	G	I	T	T	C	D	A	G	T	G	D	I	C	T	A	.	A	A	A	I	I	G	C	T	D	A
GWAS-173	49	59	G2	I	.	T	A	A	.	.	.	-	-	-	T	.	G	T	A	G	D	T	G	G	A	G	I	T	T	C	D	A	G	T	G	D	I	C	T	A	.	A	A	A	I	I	G	C	T	D	A
GWAS-167	54	65		I	.	T	A	A	.	.	.	-	-	-	T	.	G	T	A	G	D	T	G	G	A	G	I	T	T	C	D	A	G	T	G	D	I	C	T	A	.	A	A	A	I	I	G	C	T	D	A
GWAS-190	55	55		I	.	T	A	A	.	.	.	-	-	-	T	.	G	T	A	G	D	T	G	G	A	G	I	T	T	C	D	A	G	T	G	D	I	C	T	A	.	A	A	A	I	I	G	C	T	D	A
GWAS-130	59	55		I	.	T	A	A	.	.	.	-	-	-	T	.	G	T	A	G	D	T	G	G	A	G	I	T	T	C	D	A	G	T	G	D	I	C	T	A	.	A	A	A	I	I	G	C	T	D	A
GWAS-164	59	56		I	.	T	A	A	.	.	.	-	-	-	T	.	G	T	A	G	D	T	G	G	A	G	I	T	T	C	D	A	G	T	G	D	I	C	T	A	.	A	A	A	I	I	G	C	T	D	A
GWAS-170	59			I	.	T	A	A	.	.	.	-	-	-	T	.	G	T	A	G	D	T	G	G	A	G	I	T	T	C	D	A	G	T	G	D	I	C	T	A	.	A	A	A	I	I	G	C	T	D	A
GWAS-154	63	63		I	.	T	A	A	.	.	.	-	-	-	T	.	G	T	A	G	D	T	G	G	A	G	I	T	T	C	D	A	G	T	G	D	I	C	T	A	.	A	A	A	I	I	G	C	T	D	A
GWAS-159	63	69		I	.	T	A	A	.	.	.	-	-	-	T	.	G	T	A	G	D	T	G	G	A	G	I	T	T	C	D	A	G	T	G	D	I	C	T	A	.	A	A	A	I	I	G	C	T	D	A
GWAS-166	65	66		I	.	T	A	A	.	.	.	-	-	-	T	.	G	T	A	G	D	T	G	G	A	G	I	T	T	C	D	A	G	T	G	D	I	C	T	A	.	A	A	A	I	I	G	C	T	D	A
BLM25	64			.	.	.	.	.	.	.	.	.	-	-	.	C	G	T	A	G	D	T	G	G	A	G	I	T	T	C	D	A	G	T	G	D	I	C	T	A	G	A	A	A	I	I	G	C	T	D	A
GWAS-143	32	53		.	.	.	.	.	.	.	.	.	-	-	.	C	G	T	A	G	D	T	G	G	A	G	I	T	T	C	D	A	G	T	G	D	I	C	T	A	G	A	A	A	I	I	G	C	T	D	A
GWAS-142	34	61		.	.	.	.	.	.	.	.	.	-	-	.	C	G	T	A	G	D	T	G	G	A	G	I	T	T	C	D	A	G	T	G	D	I	C	T	A	G	A	A	A	I	I	G	C	T	D	A
GWAS-144	34			.	.	.	.	.	.	.	.	.	-	-	.	C	G	T	A	G	D	T	G	G	A	G	I	T	T	C	D	A	G	T	G	D	I	C	T	A	G	A	A	A	I	I	G	C	T	D	A
GWAS-176	37	67		.	.	.	.	.	.	.	.	.	-	-	.	C	G	T	A	G	D	T	G	G	A	G	I	T	T	C	D	A	G	T	G	D	I	C	T	A	G	A	A	A	I	I	G	C	T	D	A
GWAS-146	39			.	.	.	.	.	.	.	.	.	-	-	.	C	G	T	A	G	D	T	G	G	A	G	I	T	T	C	D	A	G	T	G	D	I	C	T	A	G	A	A	A	I	I	G	C	T	D	A
GWAS-189	39	60		.	.	.	.	.	.	.	.	.	-	-	.	C	G	T	A	G	D	T	G	G	A	G	I	T	T	C	D	A	G	T	G	D	I	C	T	A	G	A	A	A	I	I	G	C	T	D	A
GWAS-148	40	49		.	.	.	.	.	.	.	.	.	-	-	.	C	G	T	A	G	D	T	G	G	A	G	I	T	T	C	D	A	G	T	G	D	I	C	T	A	G	A	A	A	I	I	G	C	T	D	A
GWAS-114	41	49		.	.	.	.	.	.	.	.	.	-	-	.	C	G	T	A	G	D	T	G	G	A	G	I	T	T	C	D	A	G	T	G	D	I	C	T	A	G	A	A	A	I	I	G	C	T	D	A
GWAS-099	42	55		.	.	.	.	.	.	.	.	.	-	-	.	C	G	T	A	G	D	T	G	G	A	G	I	T	T	C	D	A	G	T	G	D	I	C	T	A	G	A	A	A	I	I	G	C	T	D	A
GWAS-139	42	49		.	.	.	.	.	.	.	.	.	-	-	.	C	G	T	A	G	D	T	G	G	A	G	I	T	T	C	D	A	G	T	G	D	I	C	T	A	G	A	A	A	I	I	G	C	T	D	A
GWAS-133	42	49		.	.	.	.	.	.	.	.	.	-	-	.	C	G	T	A	G	D	T	G	G	A	G	I	T	T	C	D	A	G	T	G	D	I	C	T	A	G	A	A	A	I	I	G	C	T	D	A
GWAS-145	42			.	.	.	.	.	.	.	.	.	-	-	.	C	G	T	A	G	D	T	G	G	A	G	I	T	T	C	D	A	G	T	G	D	I	C	T	A	G	A	A	A	I	I	G	C	T	D	A
GWAS-151	42	55		.	.	.	.	.	.	.	.	.	-	-	.	C	G	T	A	G	D	T	G	G	A	G	I	T	T	C	D	A	G	T	G	D	I	C	T	A	G	A	A	A	I	I	G	C	T	D	A
GWAS-149	43			.	.	.	.	.	.	.	.	.	-	-	.	C	G	T	A	G	D	T	G	G	A	G	I	T	T	C	D	A	G	T	G	D	I	C	T	A	G	A	A	A	I	I	G	C	T	D	A
GWAS-101	44	63		.	.	.	.	.	.	.	.	.	-	-	.	C	G	T	A	G	D	T	G	G	A	G	I	T	T	C	D	A	G	T	G	D	I	C	T	A	G	A	A	A	I	I	G	C	T	D	A
GWAS-102	44	55		.	.	.	.	.	.	.	.	.	-	-	.	C	G	T	A	G	D	T	G	G	A	G	I	T	T	C	D	A	G	T	G	D	I	C	T	A	G	A	A	A	I	I	G	C	T	D	A
GWAS-104	44	61		.	.	.	.	.	.	.	.	.	-	-	.	C	G	T	A	G	D	T	G	G	A	G	I	T	T	C	D	A	G	T	G	D	I	C	T	A	G	A	A	A	I	I	G	C	T	D	A
GWAS-107	44	53		.	.	.	.	.	.	.	.	.	-	-	.	C	G	T	A	G	D	T	G	G	A	G	I	T	T	C	D	A	G	T	G	D	I	C	T	A	G	A	A	A	I	I	G	C	T	D	A
GWAS-108	44	51		.	.	.	.	.	.	.	.	.	-	-	.	C	G	T	A	G	D	T	G	G	A	G	I	T	T	C	D	A	G	T	G	D	I	C	T	A	G	A	A	A	I	I	G	C	T	D	A
GWAS-109	44	55		.	.	.	.	.	.	.	.	.	-	-	.	C	G	T	A	G	D	T	G	G	A	G	I	T	T	C	D	A	G	T	G	D	I	C	T	A	G	A	A	A	I	I	G	C	T	D	A
GWAS-111	44	51		.	.	.	.	.	.	.	.	.	-	-	.	C	G	T	A	G	D	T	G	G	A	G	I	T	T	C	D	A	G	T	G	D	I	C	T	A	G	A	A	A	I	I	G	C	T	D	A
GWAS-126	44	55		.	.	.	.	.	.	.	.	.	-	-	.	C	G	T	A	G	D	T	G	G	A	G	I	T	T	C	D	A	G	T	G	D	I	C	T	A	G	A	A	A	I	I	G	C	T	D	A
GWAS-182	44	69		.	.	.	.	.	.	.	.	.	-	-	.	C	G	T	A	G	D	T	G	G	A	G	I	T	T	C	D	A	G	T	G	D	I	C	T	A	G	A	A	A	I	I	G	C	T	D	A
GWAS-120	46	55		.	.	.	.	.	.	.	.	.	-	-	.	C	G	T	A	G	D	T	G	G	A	G	I	T	T	C	D	A	G	T	G	D	I	C	T	A	G	A	A	A	I	I	G	C	T	D	A
GWAS-121	46	55		.	.	.	.	.	.	.	.	.	-	-	.	C	G	T	A	G	D	T	G	G	A	G	I	T	T	C	D	A	G	T	G	D	I	C	T	A	G	A	A	A	I	I	G	C	T	D	A
GWAS-110	47	53		.	.	.	.	.	.	.	.	.	-	-	.	C	G	T	A	G	D	T	G	G	A	G	I	T	T	C	D	A	G	T	G	D	I	C	T	A	G	A	A	A	I	I	G	C	T	D	A
GWAS-105	48	55		.	.	.	.	.	.	.	.	.	-	-	.	C	G	T	A	G	D	T	G	G	A	G	I	T	T	C	D	A	G	T	G	D	I	C	T	A	G	A	A	A	I	I	G	C	T	D	A
GWAS-119	48	59		.	.	.	.	.	.	.	.	.	-	-	.	C	G	T	A	G	D	T	G	G	A	G	I	T	T	C	D	A	G	T	G	D	I	C	T	A	G	A	A	A	I	I	G	C	T	D	A
GWAS-098	49	55		.	.	.	.	.	.	.	.	.	-	-	.	C	G	T	A	G	D	T	G	G	A	G	I	T	T	C	D	A	G	T	G	D	I	C	T	A	G	A	A	A	I	I	G	C	T	D	A
GWAS-100	49	69		.	.	.	.	.	.	.	.	.	-	-	.	C	G	T	A	G	D	T	G	G	A	G	I	T	T	C	D	A	G	T	G	D	I	C	T	A	G	A	A	A	I	I	G	C	T	D	A
GWAS-118	49	67		.	.	.	.	.	.	.	.	.	-	-	.	C	G	T	A	G	D	T	G	G	A	G	I	T	T	C	D	A	G	T	G	D	I	C	T	A	G	A	A	A	I	I	G	C	T	D	A
GWAS-132	49	59		.	.	.	.	.	.	.	.	.	-	-	.	C	G	T	A	G	D	T	G	G	A	G	I	T	T	C	D	A	G	T	G	D	I	C	T	A	G	A	A	A	I	I	G	C	T	D	A
GWAS-150	49	67		.	.	.	.	.	.	.	.	.	-	-	.	C	G	T	A	G	D	T	G	G	A	G	I	T	T	C	D	A	G	T	G	D	I	C	T	A	G	A	A	A	I	I	G	C	T	D	A
GWAS-152	49			.	.	.	.	.	.	.	.	.	-	-	.	C	G	T	A	G	D	T	G	G	A	G	I	T	T	C	D	A	G	T	G	D	I	C	T	A	G	A	A	A	I	I	G	C	T	D	A
GWAS-174	49	64		.	.	.	.	.	.	.	.	.	-	-	.	C	G	T	A	G	D	T	G	G	A	G	I	T	T	C	D	A	G	T	G	D	I	C	T	A	G	A	A	A	I	I	G	C	T	D	A
GWAS-177	49	55		.	.	.	.	.	.	.	.	.	-	-	.	C	G	T	A	G	D	T	G	G	A	G	I	T	T	C	D	A	G	T	G	D	I	C	T	A	G	A	A	A	I	I	G	C	T	D	A
GWAS-125	50	69		.	.	.	.	.	.	.	.	.	-	-	.	C	G	T	A	G	D	T	G	G	A	G	I	T	T	C	D	A	G	T	G	D	I	C	T	A	G	A	A	A	I	I	G	C	T	D	A
GWAS-103	51	61		.	.	.	.	.	.	.	.	.	-	-	.	C	G	T	A	G	D	T	G	G	A	G	I	T	T	C	D	A	G	T	G	D	I	C	T	A	G	A	A	A	I	I	G	C	T	D	A
GWAS-134	53	63	G3	.	.	.	.	.	.	.	.	.	-	-	.	C	G	T	A	G	D	T	G	G	A	G	I	T	T	C	D	A	G	T	G	D	I	C	T	A	G	A	A	A	I	I	G	C	T	D	A
GWAS-096	54	69		.	.	.	.	.	.	.	.	.	-	-	.	C	G	T	A	G	D	T	G	G	A	G	I	T	T	C	D	A	G	T	G	D	I	C	T	A	G	A	A	A	I	I	G	C	T	D	A
GWAS-137	54			.	.	.	.	.	.	.	.	.	-	-	.	C	G	T	A	G	D	T	G	G	A	G	I	T	T	C	D	A	G	T	G	D	I	C	T	A	G	A	A	A	I	I	G	C	T	D	A
GWAS-138	54	66		.	.	.	.	.	.	.	.	.	-	-	.	C	G	T	A	G	D	T	G	G	A	G	I	T	T	C	D	A	G	T	G	D	I	C	T	A	G	A	A	A	I	I	G	C	T	D	A
GWAS-161	54			.	.	.	.	.	.	.	.	.	-	-	.	C	G	T	A	G	D	T	G	G	A	G	I	T	T	C	D	A	G	T	G	D	I	C	T	A	G	A	A	A	I	I	G	C	T	D	A
GWAS-165	54			.	.	.	.	.	.	.	.	.	-	-	.	C	G	T	A	G	D	T	G	G	A	G	I	T	T	C	D	A	G	T	G	D	I	C	T	A	G	A	A	A	I	I	G	C	T	D	A
GWAS-178	54	64		.	.	.	.	.	.	.	.	.	-	-	.	C	G	T	A	G	D	T	G	G	A	G	I	T	T	C	D	A	G	T	G	D	I	C	T	A	G	A	A	A	I	I	G	C	T	D	A
GWAS-185	54	55		.	.	.	.	.	.	.	.	.	-	-	.	C	G	T	A	G	D	T	G	G	A	G	I	T	T	C	D	A	G	T	G	D	I	C	T	A	G	A	A	A	I	I	G	C	T	D	A
GWAS-186	54	69		.	.	.	.	.	.	.	.	.	-	-	.	C	G	T	A	G	D	T	G	G	A	G	I	T	T	C	D	A	G	T	G	D	I	C	T	A	G	A	A	A	I	I	G	C	T	D	A
GWAS-095	55	61		.	.	.	.	.	.	.	.	.	-	-	.	C	G	T	A	G	D	T	G	G	A	G	I	T	T	C	D	A	G	T	G	D	I	C	T	A	G	A	A	A	I	I	G	C	T	D	A
GWAS-117	55	63		.	.	.	.	.	.	.	.	.	-	-	.	C	G	T	A	G	D	T	G	G	A	G	I	T	T	C	D	A	G	T	G	D	I	C	T	A	G	A	A	A	I	I	G	C	T	D	A
GWAS-160	55	70		.	.	.	.	.	.	.	.	.	-	-	.	C	G	T	A	G	D	T	G	G	A	G	I	T	T	C	D	A	G	T	G	D	I	C	T	A	G	A	A	A	I	I	G	C	T	D	A
GWAS-141	56			.	.	.	.	.	.	.	.	.	-	-	.	C	G	T	A	G	D	T	G	G	A	G	I	T	T	C	D	A	G	T	G	D	I	C	T	A	G	A	A	A	I	I	G	C	T	D	A
GWAS-181	56	63		.	.	.	.	.	.	.	.	.	-	-	.	C	G	T	A	G	D	T	G	G	A	G	I	T	T	C	D	A	G	T	G	D	I	C	T	A	G	A	A	A	I	I	G	C	T	D	A
GWAS-097	57	63		.	.	.	.	.	.	.	.	.	-	-	.	C	G	T	A	G	D	T	G	G	A	G	I	T	T	C	D	A	G	T	G	D	I	C	T	A	G	A	A	A	I	I	G	C	T	D	A
GWAS-123	57	69		.	.	.	.	.	.	.	.	.	-	-	.	C	G	T	A	G	D	T	G	G	A	G	I	T	T	C	D	A	G	T	G	D	I	C	T	A	G	A	A	A	I	I	G	C	T	D	A
GWAS-135	58	64		.	.	.	.	.	.	.	.	.	-	-	.	C	G	T	A	G	D	T	G	G	A	G	I	T	T	C	D	A	G	T	G	D	I	C	T	A	G	A	A	A	I	I	G	C	T	D	A
GWAS-116	59	49		.	.	.	.	.	.	.	.	.	-	-	.	C	G	T	A	G	D	T	G	G	A	G	I	T	T	C	D	A	G	T	G	D	I	C	T	A	G	A	A	A	I	I	G	C	T	D	A
GWAS-124	59	74		.	.	.	.	.	.	.	.	.	-	-	.	C	G	T	A	G	D	T	G	G	A	G	I	T	T	C	D	A	G	T	G	D	I	C	T	A	G	A	A	A	I	I	G	C	T	D	A
GWAS-128	59	55		.	.	.	.	.	.	.	.	.	-	-	.	C	G	T	A	G	D	T	G	G	A	G	I	T	T	C	D	A	G	T	G	D	I	C	T	A	G	A	A	A	I	I	G	C	T	D	A
GWAS-129	59	55		.	.	.	.	.	.	.	.	.	-	-	.	C	G	T	A	G	D	T	G	G	A	G	I	T	T	C	D	A	G	T	G	D	I	C	T	A	G	A	A	A	I	I	G	C	T	D	A
GWAS-156	59	61		.	.	.	.	.	.	.	.	.	-	-	.	C	G	T	A	G	D	T	G	G	A	G	I	T	T	C	D	A	G	T	G	D	I	C	T	A	G	A	A	A	I	I	G	C	T	D	A
GWAS-187	59	61		.	.	.	.	.	.	.	.	.	-	-	.	C	G	T	A	G	D	T	G	G	A	G	I	T	T	C	D	A	G	T	G	D	I	C	T	A	G	A	A	A	I	I	G	C	T	D	A
GWAS-162	60	69		.	.	.	.	.	.	.	.	.	-	-	.	C	G	T	A	G	D	T	G	G	A	G	I	T	T	C	D	A	G	T	G	D	I	C	T	A	G	A	A	A	I	I	G	C	T	D	A
GWAS-112	61	61		.	.	.	.	.	.	.	.	.	-	-	.	C	G	T	A	G	D	T	G	G	A	G	I	T	T	C	D	A	G	T	G	D	I	C	T	A	G	A	A	A	I	I	G	C	T	D	A
GWAS-180	61	64		.	.	.	.	.	.	.	.	.	-	-	.	C	G	T	A	G	D	T	G	G	A	G	I	T	T	C	D	A	G	T	G	D	I	C	T	A	G	A	A	A	I	I	G	C	T	D	A
GWAS-147	62			.	.	.	.	.	.	.	.	.	-	-	.	C	G	T	A	G	D	T	G	G	A	G	I	T	T	C	D	A	G	T	G	D	I	C	T	A	G	A	A	A	I	I	G	C	T	D	A
GWAS-175	62	64		.	.	.	.	.	.	.	.	.	-	-	.	C	G	T	A	G	D	T	G	G	A	G	I	T	T	C	D	A	G	T	G	D	I	C	T	A	G	A	A	A	I	I	G	C	T	D	A
GWAS-179	62	72		.	.	.	.	.	.	.	.	.	-	-	.	C	G	T	A	G	D	T	G	G	A	G	I	T	T	C	D	A	G	T	G	D	I	C	T	A	G	A	A	A	I	I	G	C	T	D	A
GWAS-115	64	75		.	.	.	.	.	.	.	.	.	-	-	.	C	G	T	A	G	D	T	G	G	A	G	I	T	T	C	D	A	G	T	G	D	I	C	T	A	G	A	A	A	I	I	G	C	T	D	A
GWAS-131	64	63		.	.	.	.	.	.	.	.	.	-	-	.	C	G	T	A	G	D	T	G	G	A	G	I	T	T	C	D	A	G	T	G	D	I	C	T	A	G	A	A	A	I	I	G	C	T	D	A
GWAS-158	64	55		.	.	.	.	.	.	.	.	.	-	-	.	C	G	T	A	G	D	T	G	G	A	G	I	T	T	C	D	A	G	T	G	D	I	C	T	A	G	A	A	A	I	I	G	C	T	D	A
GWAS-163	64	70		.	.	.	.	.	.	.	.	.	-	-	.	C	G	T	A	G	D	T	G	G	A	G	I	T	T	C	D	A	G	T	G	D	I	C	T	A	G	A	A	A	I	I	G	C	T	D	A
GWAS-169	64	61		.	.	.	.	.	.	.	.	.	-	-	.	C	G	T	A	G	D	T	G	G	A	G	I	T	T	C	D	A	G	T	G	D	I	C	T	A	G	A	A	A	I	I	G	C	T	D	A
GWAS-171	64	64		.	.	.	.	.	.	.	.	.	-	-	.	C	G	T	A	G	D	T	G	G	A	G	I	T	T	C	D	A	G	T	G	D	I	C	T	A	G	A	A	A	I	I	G	C	T	D	A
GWAS-183	64	88		.	.	.	.	.	.	.	.	.	-	-	.	C	G	T	A	G	D	T	G	G	A	G	I	T	T	C	D	A	G	T	G	D	I	C	T	A	G	A	A	A	I	I	G	C	T	D	A
GWAS-136	68	69		.	.	.	.	.	.	.	.	.	-	-	.	C	G	T	A	G	D	T	G	G	A	G	I	T	T	C	D	A	G	T	G	D	I	C	T	A	G	A	A	A	I	I	G	C	T	D	A
GWAS-157	69	61		.	.	.	.	.	.	.	.	.	-	-	.	C	G	T	A	G	D	T	G	G	A	G	I	T	T	C	D	A	G	T	G	D	I	C	T	A	G	A	A	A	I	I	G	C	T	D	A
GWAS-113	70	75		.	.	.	.	.	.	.	.	.	-	-	.	C	G	T	A	G	D	T	G	G	A	G	I	T	T	C	D	A	G	T	G	D	I	C	T	A	G	A	A	A	I	I	G	C	T	D	A
GWAS-127	76	72		.	.	.	.	.	.	.	.	.	-	-	.	C	G	T	A	G	D	T	G	G	A	G	I	T	T	C	D	A	G	T	G	D	I	C	T	A	G	A	A	A	I	I	G	C	T	D	A

Supplementary Figure 4 (to be continued)

b

PAN	DCI			exon1	intron3					exon4	intron4								exon5		intron5					exon6	exon8
	2014	2015		43	455	486	492	498	506	636-641	897	994-995	1080	1100	1113	1210	1235	1246	1344	1376	1397	1419	1425	1433	1463	1485	1792
reference				T	A	T	-	T	G	-	T	TT	T	-	G	T	C	A	A	T	A	A	T	A	A	T	C
BLM29				.	-	-	T	A	.	.	.	AA	A	T	A	C	G	.	C	A	T	G	-	-	G	G	A
GWAS-191	16			.	-	.	T	-	.	GAGGAG	C	AA	A	T	A	C	G	G	C	.	T	G	.	.	.	.	A
GWAS-192	16	48		.	-	-	T	A	.	.	.	AA	A	T	A	C	G	.	C	A	T	G	-	-	G	G	A
GWAS-202	26	57		.	-	-	T	A	.	.	.	AA	A	T	A	C	G	.	C	A	T	G	-	-	G	G	A
GWAS-195	31	48		.	.	.	T	-	.	GAGGAG	C	AA	A	T	A	C	G		C	.	T	G	.	.	.	.	A
GWAS-206	31	54		.	-	.	T	-	.	GAGGAG	C	AA	A	T	A	C	G	G	C	.	T	G	.	.	.	.	A
GWAS-207	31	38		.	-	.	T	-	.	GAGGAG	C	AA	A	T	A	C	G	G	C	.	T	G	.	.	.	.	A
GWAS-200	36	57		.	-	.	T	-	.	GAGGAG	C	AA	A	T	A	C	G	G	C	.	T	G	.	.	.	.	A
GWAS-201	36	38	G1	.	-	.	T	-	.	GAGGAG	C	AA	A	T	A	C	G	G	C	.	T	G	.	.	.	.	A
GWAS-203	36	50		.	-	.	T	-	.	GAGGAG	C	AA	A	T	A	C	G	G	C	.	T	G	.	.	.	.	A
GWAS-204	36	50		.	-	.	T	-	.	GAGGAG	C	AA	A	T	A	C	G	G	C	.	T	G	.	.	.	.	A
GWAS-205	36	57		.	-	.	T	-	.	GAGGAG	C	AA	A	T	A	C	G	G	C	.	T	G	.	.	.	.	A
GWAS-196	41	38		.	-	.	T	-	.	GAGGAG	C	AA	A	T	A	C	G	G	C	.	T	G	.	.	.	.	A
GWAS-197	41	38		.	-	.	T	-	.	GAGGAG	C	AA	A	T	A	C	G	G	C	.	T	G	.	.	.	.	A
GWAS-193	61	62		.	-	.	T	-	.	GAGGAG	C	AA	A	T	A	C	G	G	C	.	T	G	.	.	.	.	A
GWAS-198	66			.	-	.	T	A	.	.	.	AA	A	T	A	C	G	G	C	.	T	G	.	.	.	.	A
GWAS-199	66			.	-	-	T	-	.	GAGGAG		AA	A	T	A	C	G	.	C	A	T	G	-	-	G	G	A
GWAS-194	71			.	.	.	T	-	.	.	.	.	A	T	A	C	G	G	C	A	T	G	-	-	G	G	A
BLM25	64			C	.	.	A	A	A	.	.	.	.	.	.	.	.	.	.	.	.	.	.	.	.	.	.
GWAS-143	32	53		C	.	.	A	A	A	.	.	.	.	.	.	.	.	.	.	.	.	.	.	.	.	.	.
GWAS-142	34	61		C	.	.	A	A	A	.	.	.	.	.	.	.	.	.	.	.	.	.	.	.	.	.	.
GWAS-144	34			C	.	.	A	A	A	.	.	.	.	.	.	.	.	.	.	.	.	.	.	.	.	.	.
GWAS-172	35	61		C	.	.	A	A	A	.	.	.	.	.	.	.	.	.	.	.	.	.	.	.	.	.	.
GWAS-176	37	67		.	.	.	T	-	.	.	.	.	.	.	.	C	.	.	.	.	.	.	.	.	.	.	A
GWAS-146	39	56		C	.	.	A	A	A	.	.	.	.	.	.	.	.	.	.	.	.	.	.	.	.	.	.
GWAS-168	39			.	-	-	T	A	.	.	.	AA	A	.	.	C	.	.	.	.	.	.	.	.	.	.	A
GWAS-189	39	60		.	.	.	T	-	.	.	.	.	.	.	.	C	.	.	.	.	.	.	.	.	.	.	A
GWAS-148	40	61		C	.	.	A	A	A	.	.	.	.	.	.	.	.	.	.	.	.	.	.	.	.	.	.
GWAS-184	40	49		.	.	.	T	-	.	.	.	.	.	.	.	C	.	.	.	.	.	.	.	.	.	.	A
GWAS-114	41	49		C	.	.	A	A	A	.	.	.	.	.	.	.	.	.	.	.	.	.	.	.	.	.	.
GWAS-099	42	55		C	.	.	A	A	A	.	.	.	.	.	.	.	.	.	.	.	.	.	.	.	.	.	.
GWAS-133	42	49		C	.	.	A	A	A	.	.	.	.	.	.	.	.	.	.	.	.	.	.	.	.	.	.
GWAS-139	42	49		C	.	.	A	A	A	.	.	.	.	.	.	.	.	.	.	.	.	.	.	.	.	.	.
GWAS-145	42			C	.	.	A	A	A	.	.	.	.	.	.	.	.	.	.	.	.	.	.	.	.	.	.
GWAS-151	42	55		C	.	.	A	A	A	.	.	.	.	.	.	.	.	.	.	.	.	.	.	.	.	.	.
GWAS-149	43	52		C	.	.	A	A	A	.	.	.	.	.	.	.	.	.	.	.	.	.	.	.	.	.	.
GWAS-153	43			C	.	.	A	A	A	.	.	.	.	.	.	.	.	.	.	.	.	.	.	.	.	.	.
GWAS-101	44	59		C	.	.	A	A	A	.	.	.	.	.	.	.	.	.	.	.	.	.	.	.	.	.	.
GWAS-102	44	56		C	.	.	A	A	A	.	.	.	.	.	.	.	.	.	.	.	.	.	.	.	.	.	.
GWAS-104	44	67		C	.	.	A	A	A	.	.	.	.	.	.	.	.	.	.	.	.	.	.	.	.	.	.
GWAS-107	44	63		C	.	.	A	A	A	.	.	.	.	.	.	.	.	.	.	.	.	.	.	.	.	.	.
GWAS-108	44	55		C	.	.	A	A	A	.	.	.	.	.	.	.	.	.	.	.	.	.	.	.	.	.	.
GWAS-109	44	61		C	.	.	A	A	A	.	.	.	.	.	.	.	.	.	.	.	.	.	.	.	.	.	.
GWAS-111	44	53		C	.	.	A	A	A	.	.	.	.	.	.	.	.	.	.	.	.	.	.	.	.	.	.
GWAS-122	44	51		.	.	.	T	-	.	.	.	.	.	.	.	C	.	.	.	.	.	.	.	.	.	.	A
GWAS-126	44	55		.	.	.	T	-	.	.	.	.	.	.	.	C	.	.	.	.	.	.	.	.	.	.	A
GWAS-155	44	51		.	.	.	T	-	.	.	.	.	.	.	.	C	.	.	.	.	.	.	.	.	.	.	A
GWAS-182	44	55		C	.	.	A	A	A	.	.	.	.	.	.	.	.	.	.	.	.	.	.	.	.	.	.
GWAS-188	44	69		C	.	.	A	A	A	.	.	.	.	.	.	.	.	.	.	.	.	.	.	.	.	.	.
GWAS-120	46	55		C	.	.	A	A	A	.	.	.	.	.	.	.	.	.	.	.	.	.	.	.	.	.	.
GWAS-121	46	55		C	.	.	A	A	A	.	.	.	.	.	.	.	.	.	.	.	.	.	.	.	.	.	.
GWAS-110	47	53		C	.	.	A	A	A	.	.	.	.	.	.	.	.	.	.	.	.	.	.	.	.	.	.
GWAS-105	48	55		C	.	.	A	A	A	.	.	.	.	.	.	.	.	.	.	.	.	.	.	.	.	.	.
GWAS-119	48	59		C	.	.	A	A	A	.	.	.	.	.	.	.	.	.	.	.	.	.	.	.	.	.	.
GWAS-098	49	49		C	.	.	A	A	A	.	.	.	.	.	.	.	.	.	.	.	.	.	.	.	.	.	.
GWAS-100	49	59		C	.	.	A	A	A	.	.	.	.	.	.	.	.	.	.	.	.	.	.	.	.	.	.
GWAS-106	49	55		C	.	.	A	A	A	.	.	.	.	.	.	.	.	.	.	.	.	.	.	.	.	.	.
GWAS-118	49	69		C	.	.	A	A	A	.	.	.	.	.	.	.	.	.	.	.	.	.	.	.	.	.	.
GWAS-132	49	67		C	.	.	A	A	A	.	.	.	.	.	.	.	.	.	.	.	.	.	.	.	.	.	.
GWAS-150	49	59		C	.	.	A	A	A	.	.	.	.	.	.	.	.	.	.	.	.	.	.	.	.	.	.
GWAS-152	49	67		C	.	.	A	A	A	.	.	.	.	.	.	.	.	.	.	.	.	.	.	.	.	.	.
GWAS-173	49			.	.	.	T	-	.	.	.	.	.	.	.	C	.	.	.	.	.	.	.	.	.	.	A
GWAS-174	49	64		.	.	.	T	-	.	.	.	.	.	.	.	C	.	.	.	.	.	.	.	.	.	.	A
GWAS-177	49	55		.	.	.	T	-	.	.	.	.	.	.	.	C	.	.	.	.	.	.	.	.	.	.	A
GWAS-125	50	69		C	.	.	A	A	A	.	.	.	.	.	.	.	.	.	.	.	.	.	.	.	.	.	.
GWAS-103	51	61	G2	C	.	.	A	A	A	.	.	.	.	.	.	.	.	.	.	.	.	.	.	.	.	.	.
GWAS-134	53	63		C	.	.	A	A	A	.	.	.	.	.	.	.	.	.	.	.	.	.	.	.	.	.	.
GWAS-096	54	65		.	.	.	T	-	.	.	.	.	.	.	.	C	.	.	.	.	.	.	.	.	.	.	A
GWAS-137	54	69		C	.	.	A	A	A	.	.	.	.	.	.	.	.	.	.	.	.	.	.	.	.	.	.
GWAS-138	54			C	.	.	A	A	A	.	.	.	.	.	.	.	.	.	.	.	.	.	.	.	.	.	.
GWAS-161	54	66		C	.	.	A	A	A	.	.	.	.	.	.	.	.	.	.	.	.	.	.	.	.	.	.
GWAS-165	54			.	.	.	T	-	.	.	.	.	.	.	.	C	.	.	.	.	.	.	.	.	.	.	A
GWAS-167	54			.	.	.	T	-	.	.	.	.	.	.	.	C	.	.	.	.	.	.	.	.	.	.	A
GWAS-178	54	64		.	.	.	T	-	.	.	.	.	.	.	.	C	.	.	.	.	.	.	.	.	.	.	A
GWAS-185	54	55		.	.	.	T	-	.	.	.	.	.	.	.	C	.	.	.	.	.	.	.	.	.	.	A
GWAS-186	54	69		C	.	.	A	A	A	.	.	.	.	.	.	.	.	.	.	.	.	.	.	.	.	.	.
GWAS-95	55	55		C	.	.	A	A	A	.	.	.	.	.	.	.	.	.	.	.	.	.	.	.	.	.	.
GWAS-117	55	61		C	.	.	A	A	A	.	.	.	.	.	.	.	.	.	.	.	.	.	.	.	.	.	.
GWAS-160	55	63		.	.	.	T	-	.	.	.	.	.	.	.	C	.	.	.	.	.	.	.	.	.	.	A
GWAS-190	55	70		.	.	.	T	-	.	.	.	.	.	.	.	C	.	.	.	.	.	.	.	.	.	.	A
GWAS-141	56			.	.	.	T	-	.	.	.	.	.	.	.	C	.	.	.	.	.	.	.	.	.	.	A
GWAS-181	56	63		.	.	.	T	-	.	.	.	.	.	.	.	C	.	.	.	.	.	.	.	.	.	.	A
GWAS-097	57	63		C	.	.	A	A	A	.	.	.	.	.	.	.	.	.	.	.	.	.	.	.	.	.	.
GWAS-123	57	69		C	.	.	A	A	A	.	.	.	.	.	.	.	.	.	.	.	.	.	.	.	.	.	.
GWAS-135	58	64		C	.	.	A	A	A	.	.	.	.	.	.	.	.	.	.	.	.	.	.	.	.	.	.
GWAS-116	59	55		C	.	.	A	A	A	.	.	.	.	.	.	.	.	.	.	.	.	.	.	.	.	.	.
GWAS-124	59	56		C	.	.	A	A	A	.	.	.	.	.	.	.	.	.	.	.	.	.	.	.	.	.	.
GWAS-128	59			.	.	.	T	-	.	.	.	.	.	.	.	C	.	.	.	.	.	.	.	.	.	.	A
GWAS-129	59	49		C	.	.	A	A	A	.	.	.	.	.	.	.	.	.	.	.	.	.	.	.	.	.	.
GWAS-130	59	74		C	.	.	A	A	A	.	.	.	.	.	.	.	.	.	.	.	.	.	.	.	.	.	.
GWAS-156	59	55		C	.	.	A	A	A	.	.	.	.	.	.	.	.	.	.	.	.	.	.	.	.	.	.
GWAS-164	59	55		.	.	.	T	-	.	.	.	.	.	.	.	C	.	.	.	.	.	.	.	.	.	.	A
GWAS-170	59	61		C	.	.	A	A	A	.	.	.	.	.	.	.	.	.	.	.	.	.	.	.	.	.	.
GWAS-187	59	61		C	.	.	A	A	A	.	.	.	.	.	.	.	.	.	.	.	.	.	.	.	.	.	.
GWAS-162	60	69		.	.	.	T	-	.	.	.	.	.	.	.	C	.	.	.	.	.	.	.	.	.	.	A
GWAS-112	61	61		C	.	.	A	A	A	.	.	.	.	.	.	.	.	.	.	.	.	.	.	.	.	.	.
GWAS-180	61	64		.	.	.	T	-	.	.	.	.	.	.	.	C	.	.	.	.	.	.	.	.	.	.	A
GWAS-147	62			C	.	.	A	A	A	.	.	.	.	.	.	.	.	.	.	.	.	.	.	.	.	.	.
GWAS-175	62	64		.	.	.	T	-	.	.	.	.	.	.	.	C	.	.	.	.	.	.	.	.	.	.	A
GWAS-179	62	72		.	.	.	T	-	.	.	.	.	.	.	.	C	.	.	.	.	.	.	.	.	.	.	A
GWAS-154	63	63		.	.	.	T	-	.	.	.	.	.	.	.	C	.	.	.	.	.	.	.	.	.	.	A
GWAS-159	63	69		C	.	.	A	A	A	.	.	.	.	.	.	.	.	.	.	.	.	.	.	.	.	.	.
GWAS-115	64	75		C	.	.	A	A	A	.	.	.	.	.	.	.	.	.	.	.	.	.	.	.	.	.	.
GWAS-131	64	63		C	.	.	A	A	A	.	.	.	.	.	.	.	.	.	.	.	.	.	.	.	.	.	.
GWAS-158	64	55		C	.	.	A	A	A	.	.	.	.	.	.	.	.	.	.	.	.	.	.	.	.	.	.
GWAS-163	64	70		.	.	.	T	-	.	.	.	.	.	.	.	C	.	.	.	.	.	.	.	.	.	.	A
GWAS-169	64	61		.	.	.	T	-	.	.	.	.	.	.	.	C	.	.	.	.	.	.	.	.	.	.	A
GWAS-171	64	64		.	.	.	T	-	.	.	.	.	.	.	.	C	.	.	.	.	.	.	.	.	.	.	A
GWAS-183	64	88		.	.	.	T	-	.	.	.	.	.	.	.	C	.	.	.	.	.	.	.	.	.	.	A
GWAS-166	65	66		C	.	.	A	A	A	.	.	.	.	.	.	.	.	.	.	.	.	.	.	.	.	.	.
GWAS-136	68	69		C	.	.	A	A	A	.	.	.	.	.	.	.	.	.	.	.	.	.	.	.	.	.	.
GWAS-157	69	61		.	.	.	T	-	.	.	.	.	.	.	.	C	.	.	.	.	.	.	.	.	.	.	A
GWAS-113	70	75		C	.	.	A	A	A	.	.	.	.	.	.	.	.	.	.	.	.	.	.	.	.	.	.
GWAS-127	76	72		C	.	.	A	A	A	.	.	.	.	.	.	.	.	.	.	.	.	.	.	.	.	.	.

Supplementary Figure 4 (to be continued)

VRN2	DCI			intron1	intron2	intron3	intron5		exon6	exon8	intron8	intron12	exon14	
	2014	2015		142	261	447	698	700	783	1062	1214	2108	2399	2402-2407
reference				C	A	A	C	G	A	C	A	C	G	.
BLM29			G1	T	.	.	.	.	G	G	T	A	A	ACAAGA
GWAS-206	31	54		T	T	G	T	T	G	.	.	.	.	.
GWAS-172	35	61		.	.	G	.	.	G	G	T	A	A	ACAAGA
GWAS-203	36	50		T	T	G	T	T	G	.	.	.	.	.
GWAS-204	36	50		T	T	G	T	T	G	.	.	.	.	.
GWAS-176	37	67		T	.	G	.	.	G	G	T	A	A	ACAAGA
GWAS-168	39	56		T	.	G	.	.	G	G	T	A	A	ACAAGA
GWAS-196	41	38		T	T	G	T	T	G	.	.	.	.	.
GWAS-153	43	52		T	.	G	.	.	G	G	T	A	A	ACAAGA
GWAS-104	44	61		T	.	.	.	.	G	G	T	A	A	ACAAGA
GWAS-155	44	56		T	.	G	.	.	G	G	T	A	A	ACAAGA
GWAS-177	49	55		T	.	G	.	.	G	G	.	.	.	.
GWAS-165	54			T	.	G	.	.	G	G	T	A	A	ACAAGA
GWAS-167	54	65		T	.	G	.	.	G	G	T	A	A	ACAAGA
GWAS-185	54	55		T	.	G	.	.	G	G	T	A	A	ACAAGA
GWAS-160	55	70		T	.	G	.	.	G	G	T	A	A	ACAAGA
GWAS-181	56	63		T	.	G	.	.	G	G	T	A	A	ACAAGA
GWAS-130	59	55		T	.	.	.	.	G	G	T	A	A	ACAAGA
GWAS-170	59			T	.	G	.	.	G	G	T	A	A	ACAAGA
GWAS-187	59	61		T	.	G	.	.	G	.	.	A	A	ACAAGA
GWAS-175	62	64		T	.	G	.	.	G	G	T	A	A	ACAAGA
GWAS-179	62	72		T	.	G	.	.	G	G	T	A	A	ACAAGA
GWAS-169	64	61		T	.	G	.	.	G	G	T	A	A	ACAAGA
GWAS-171	64	64		T	.	G	.	.	G	G	T	.	A	ACAAGA
GWAS-198	66			T	T	G	T	T	G	.	.	.	.	.
GWAS-199	66			T	T	.	T	T	G	.	.	.	.	.
BLM25	64			.	.	.	.	.	.	.	.	.	.	.
GWAS-191	16			.	.	.	.	.	.	.	.	.	.	.
GWAS-192	16	48		.	.	.	.	.	.	.	T	A	.	.
GWAS-202	26	57		.	.	.	.	.	.	.	.	.	.	.
GWAS-195	31	48		.	.	.	.	.	G	.	.	.	.	.
GWAS-207	31	38		.	.	.	.	.	.	.	.	.	.	.
GWAS-143	32	53		.	.	.	.	.	.	.	.	.	.	.
GWAS-142	34	61		.	.	.	.	.	.	.	.	.	.	.
GWAS-144	34			.	.	.	.	.	.	.	.	.	.	.
GWAS-200	36	57		.	.	.	.	.	.	.	.	.	.	.
GWAS-201	36	38		.	.	.	.	.	.	.	.	.	.	.
GWAS-205	36	57		.	.	.	.	.	.	.	.	.	.	.
GWAS-146	39			.	.	.	.	.	.	.	.	.	.	.
GWAS-189	39	60		.	.	.	.	.	.	.	.	.	.	.
GWAS-148	40	49		.	.	.	.	.	.	.	.	.	.	.
GWAS-184	40	61		.	.	.	.	.	.	.	.	.	.	.
GWAS-114	41	49		.	.	.	.	.	.	.	.	.	.	.
GWAS-197	41	38		.	.	.	.	.	.	.	.	.	.	.
GWAS-99	42	55		.	.	.	.	.	.	.	.	.	.	.
GWAS-133	42	49		.	.	.	.	.	.	.	.	.	.	.
GWAS-139	42	49		.	.	.	.	.	.	.	.	.	.	.
GWAS-145	42			.	.	.	.	.	.	.	.	.	.	.
GWAS-151	42	55		.	.	.	.	.	.	.	.	.	.	.
GWAS-149	43			.	.	.	.	.	.	.	.	.	.	.
GWAS-101	44	63		.	.	.	.	.	.	.	.	.	.	.
GWAS-102	44	55		.	.	.	.	.	.	.	.	.	.	.
GWAS-107	44	53		.	.	.	.	.	.	.	.	.	.	.
GWAS-108	44	51		.	.	.	.	.	.	.	.	.	.	.
GWAS-109	44	55		.	.	.	.	.	.	.	.	.	.	.
GWAS-111	44	51		.	.	.	.	.	.	.	.	.	.	.
GWAS-122	44	59		.	.	.	.	.	.	.	.	.	.	.
GWAS-126	44	55		.	.	.	.	.	.	.	.	.	.	.
GWAS-182	44	69		.	.	.	.	.	.	.	.	.	.	.
GWAS-188	44	67		.	.	.	.	.	.	.	.	.	.	.
GWAS-120	46	55		.	.	.	.	.	.	.	.	.	.	.
GWAS-121	46	55		.	.	.	.	.	.	.	.	.	.	.
GWAS-110	47	53		.	.	.	.	.	.	.	.	.	.	.
GWAS-105	48	55		.	.	.	.	.	.	.	.	.	.	.
GWAS-119	48	59		.	.	.	.	.	.	.	.	.	.	.
GWAS-098	49	55		.	.	.	.	.	.	.	.	.	.	.
GWAS-100	49	69		.	.	.	.	.	.	.	.	.	.	.
GWAS-106	49	49		.	.	.	.	.	.	.	.	.	.	.
GWAS-118	49	67		.	.	.	.	.	.	.	.	.	.	.
GWAS-132	49	59	G2	.	.	.	.	.	.	.	.	.	.	.
GWAS-150	49	67		.	.	.	.	.	.	.	.	.	.	.
GWAS-152	49			.	.	.	.	.	.	.	.	.	.	.
GWAS-173	49	59		.	.	.	.	.	.	.	.	.	.	.
GWAS-174	49	64		.	.	.	.	.	.	.	.	.	.	.
GWAS-125	50	69		.	.	.	.	.	.	.	.	.	.	.
GWAS-103	51	61		.	.	.	.	.	.	.	.	.	.	.
GWAS-134	53	63		.	.	.	.	.	.	.	.	.	.	.
GWAS-96	54	69		.	.	.	.	.	.	.	.	.	.	.
GWAS-137	54			.	.	.	.	.	.	.	.	.	.	.
GWAS-138	54	66		.	.	.	.	.	.	.	.	.	.	.
GWAS-161	54			.	.	.	.	.	.	.	.	.	.	.
GWAS-178	54	64		.	.	.	.	.	.	.	.	.	.	.
GWAS-186	54	69		.	.	.	.	.	.	.	.	.	.	.
GWAS-95	55	61		.	.	.	.	.	.	.	.	.	.	.
GWAS-117	55	63		.	.	.	.	.	.	.	.	.	.	.
GWAS-190	55	55		.	.	.	.	.	.	.	.	.	.	.
GWAS-141	56			.	.	.	.	.	.	.	.	.	.	.
GWAS-97	57	63		.	.	.	.	.	.	.	.	.	.	.
GWAS-123	57	69		.	.	.	.	.	.	.	.	.	.	.
GWAS-135	58	64		.	.	.	.	.	.	.	.	.	.	.
GWAS-116	59	49		.	.	.	.	.	.	.	.	.	.	.
GWAS-124	59	74		.	.	.	.	.	.	.	.	.	.	.
GWAS-128	59	55		.	.	.	.	.	.	.	.	.	.	.
GWAS-129	59	55		.	.	.	.	.	.	.	.	.	.	.
GWAS-156	59	61		T	.	.	.	.	.	.	.	A	A	ACAAGA
GWAS-164	59	56		.	.	.	.	.	.	.	.	.	.	.
GWAS-162	60	69		.	.	.	.	.	.	.	.	.	.	.
GWAS-180	61	64		.	.	.	.	.	.	.	.	.	.	.
GWAS-193	61	62		.	.	.	.	.	.	.	.	.	.	.
GWAS-147	62			.	.	.	.	.	.	.	.	.	.	.
GWAS-154	63	63		.	.	.	.	.	.	.	.	.	.	.
GWAS-159	63	69		.	.	.	.	.	.	.	.	.	.	.
GWAS-112	64	61		.	.	.	.	.	.	.	.	.	.	.
GWAS-115	64	75		.	.	.	.	.	.	.	.	.	.	.
GWAS-131	64	63		.	.	.	.	.	.	.	.	.	.	.
GWAS-158	64	55		.	.	.	.	.	.	.	.	.	.	.
GWAS-163	64	70		.	.	.	.	.	.	.	.	.	.	.
GWAS-183	64	68		.	.	.	.	.	.	.	.	.	.	.
GWAS-166	65	66		.	.	.	.	.	.	.	.	.	.	.
GWAS-136	68	69		.	.	.	.	.	.	.	.	.	.	.
GWAS-157	69	61		.	.	.	.	.	.	.	.	.	.	.
GWAS-113	70	75		.	.	.	.	.	.	.	.	.	.	.
GWAS-194	71			.	.	.	T	T	G	.	.	.	.	.
GWAS-127	76	72		.	.	.	.	.	.	.	.	.	.	.

Supplementary Figure 4 DCIs and genotypes of 112 advanced broccoli breeding lines. Three, two, and two haplotypes of *BoFLC3* (**a**), *PAN* (**b**), and *VRN2* (**c**) are classified, respectively. Different haplotypes were highlighted different colors, and different nucleotides were indicated, too.

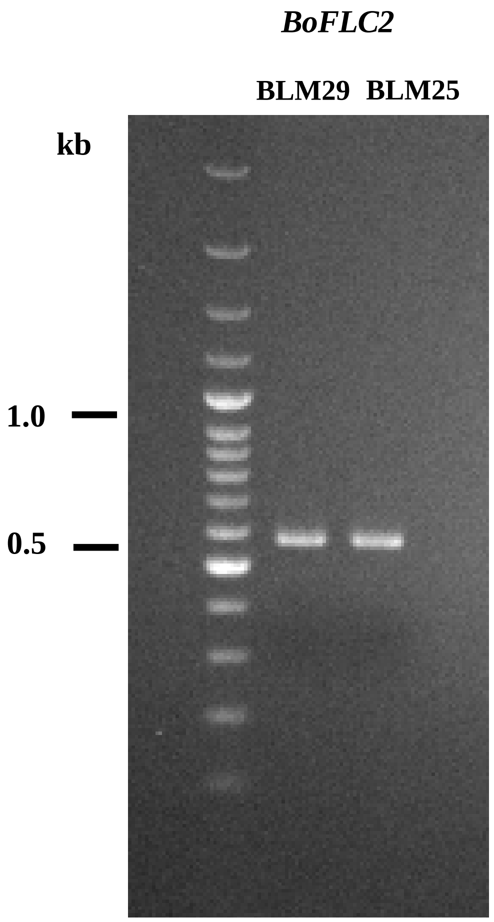

Supplementary Figure 5 The genotype of *BoFLC2* in BLM29 and BLM25. Both BLM29 and BLM25 have the same *BoFLC2* genotype. *BoFLC2* were amplified using gene-specific primers, *BoFLC2*-F (5-AGGGCCTAGAGGGCATACAT-3) and *BoFLC2*-R (5-TTTTGAGGCTCTCGACACAA-3), which were designed to cover the frameshift mutation of exon 4. The PCR products of BLM29 and BLM25, 571 bp, cannot be digested by *Alu*I because of the same 1-bp, G, deletion. The molecular weight marker is 100-bp ladder.
